# Improved glucose handling in female rat offspring of a hypertensive pregnancy with intrauterine growth restriction

**DOI:** 10.14814/phy2.70222

**Published:** 2025-02-04

**Authors:** Melissa A. Cedars, Kate M. Root, Brian Akhaphong, Megan Beetch, Abigail E. Miles, Ronald R. Regal, Emilyn U. Alejandro, Jean F. Regal

**Affiliations:** ^1^ Department of Biomedical Sciences University of Minnesota Medical School Duluth Minnesota USA; ^2^ Department of Integrative Biology and Physiology University of Minnesota Medical School Minneapolis Minnesota USA; ^3^ Department of Mathematics and Statistics University of Minnesota Duluth Minnesota USA

**Keywords:** Beta cell, developmental programming, gestational hypertension, glucose intolerance, incretins, intrauterine growth restriction

## Abstract

Hypertensive disorders of pregnancy, intrauterine growth restriction (IUGR), and reduced pancreatic β‐cell area increases risk of offspring developing type 2 diabetes (T2D). Our previous studies using rat reduced uteroplacental perfusion pressure (RUPP) model of gestational hypertension and IUGR demonstrated reduced pancreatic β‐cell area in offspring at embryonic day 19 and postnatal day 13 (PD13). We hypothesized reduced β‐cell area early in life would manifest as hyperglycemia and glucose intolerance as animals aged. However, glucose intolerance did not differ in RUPP versus control offspring to 1 year of life, whether intraperitoneal or oral glucose challenge. At PD28, female RUPP offspring show normalized β‐cell area compared to controls and improved ability to clear glucose following oral challenge. Oral glucose challenge results in significant increase in incretin GLP‐1 in RUPP female offspring compared to control. Insulin tolerance did not differ amongst control and RUPP offspring, except at PD28 where insulin reduced blood glucose more effectively in RUPP female offspring versus control. Insulin‐induced vasodilation in isolated aorta and insulin signaling in fat are more pronounced in RUPP PD28 female offspring versus control. Thus, our studies demonstrate compensatory mechanisms protect IUGR offspring of a hypertensive pregnancy from long‐term metabolic effects and development of T2D.

## 1 INTRODUCTION

Hypertensive disorders of pregnancy, including gestational hypertension and preeclampsia, can have negative outcomes for offspring including intrauterine growth restriction (IUGR). Hypertensive disorders of pregnancy and IUGR are known to increase the risk of developing type 2 diabetes (T2D) later in life (Kajantie et al., 2017; Paramsothy et al., 2023; Rashid et al., 2018; Wang et al., 2016; Zhao et al., 2018). Approximately one in 10 adults in the United States have T2D with the number of diagnoses doubling in the last 20 years. The area of the insulin‐producing β‐cells in the pancreatic islets is set early in life and sustained reduction of β‐cell area can influence the development of T2D. Thus, understanding how reduced β‐cell area in early life leads to glucose intolerance and the potential development of type 2 diabetes may shed light on strategies to improve health outcomes for offspring of hypertensive pregnancies.

Our previous studies have used the rat reduced uteroplacental perfusion pressure (RUPP) model to investigate consequences of high blood pressure and IUGR on pancreatic development and function in offspring of hypertensive pregnancies. In this model, blood flow to the placenta is surgically compromised with partial ligation of the blood supply to the uteroplacental unit at the beginning of the third trimester at gestation day (GD) 14, leading to high blood pressure in the dam and reduced fetal weight (Lillegard et al., 2013). We have demonstrated that RUPP pregnancy results in reduced pancreatic β‐cell area in the female RUPP offspring at embryonic (e) 19 and postnatal day (PD) 13 (Akhaphong et al., 2018; Root et al., 2022). Elimination of macrophages postnatally using liposomal clodronate restored β‐cell area at PD13 in RUPP offspring but did not influence glucose phenotypes when rats were 6–8 weeks of age (Root et al., 2022). Previous studies had demonstrated that offspring of RUPP pregnancies have glucose intolerance, whether IV glucose administered at 9 weeks (Heltemes et al., 2010), or oral glucose at 6–12 months (Intapad et al., 2016), but these studies did not evaluate β‐cell area in the pancreas. Thus, we hypothesized that reductions in β‐cell area which we reported on e19 and PD13 in female RUPP offspring are associated with impaired glucose handling as female offspring age. However, as reported here, we have not demonstrated glucose intolerance in RUPP offspring, either male or female. In fact, our data show that by PD28, the β‐cell area has returned to normal in female RUPP offspring, and these offspring are better able to stabilize glucose levels following oral glucose challenge and are more sensitive to insulin challenge than Sham offspring. The change in glucose tolerance following oral glucose administration led us to investigate a potential role for incretins. The incretin effect, first described in 1964, refers to the greater increase in insulin secretion after an oral glucose load when compared to an equivalent IV bolus. Since that time, GLP‐1 and GIP have been widely described as the incretins secreted by cells in the GI tract following oral glucose, and they have wide‐reaching effects beyond increasing insulin secretion. Thus, our continued studies investigated compensatory mechanisms in the female offspring that would help re‐establish glucose control following an early life reduction in β‐cell area. This includes evaluating recruitment of the incretin pathway, increased insulin‐induced vasodilation, and/or increased peripheral insulin sensitivity.

## 2 MATERIALS AND METHODS

### 2.1 Reduced uteroplacental perfusion pressure (RUPP) model of placental ischemia‐induced hypertension

Timed‐pregnant Sprague–Dawley rats (CD IGS strain; Charles River Laboratories, Raleigh, NC) with specified breeding weights of 215–225 g were housed singly in a temperature‐controlled facility, 12‐h light/dark cycle with tap drinking water and standard rat chow (Purina LabDiet 5001) ad libitum. All procedures were approved by Institutional Animal Care and Use Committee of University of Minnesota. RUPP procedures and control Sham surgeries were conducted on GD14 as previously described (Lillegard et al., 2013) with GD0 defined as date of vaginal plug. Our previous studies (Laule et al., 2017, 2019; Lillegard et al., 2013, 2014; Regal et al., 2015, 2016) have consistently shown increased blood pressure in RUPP dams with this method. In current experiments, blood pressure was not monitored. Pups were weighed within 24 h of birth and litters culled to eight pups with preference for females. Pups were housed with birth mother until weaning at PD21. Weaned pups of the same sex and age were housed together until euthanized.

Dams were randomly assigned to either a RUPP or Sham surgery group, and pups were randomly assigned to glucose phenotype treatments and identified by number so assessment was blinded to the operator. Number of animals was determined by power analysis using variances obtained from previous glucose phenotype experiments in the lab.

### 2.2 Experimental design

**FIGURE 1:**
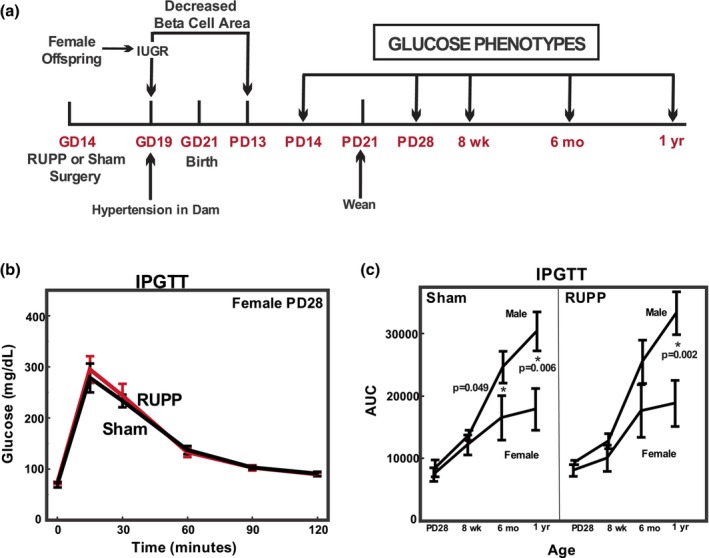
Glucose phenotype testing of offspring following maternal Reduced Uteroplacental Perfusion Pressure (RUPP) surgery. (a) Timed‐pregnant Sprague–Dawley dams underwent either RUPP or Sham surgery on gestation day (GD) 14. Dams gave birth on approximately GD21 and pups were weaned at postnatal day (PD) 21. Glucose phenotypes were assessed at PD14, PD28, 8 weeks, 6 months, and 1 year as described in Methods. (b) Intraperitoneal glucose tolerance testing (IPGTT) of PD28 female offspring from RUPP and Sham pregnancies. Glucose was determined by glucometer in 6–10 offspring from different litters. (c) Area under the curve (AUC) after IPGTT of male and female offspring of varying ages. AUC from time 0 glucose was calculated after IP glucose challenge using the 30, 60, 90, and 120 min time points for all ages and both sexes as seen in Figure S3. Values represent mean +/− SE from 6 to 11 offspring of each sex from different litters. ANOVA detected a significant sex (*p* = 0.0001) and age (*p* = 0.0001) effect overall. *p values less than 0.05 are indicated for individual sex contrast following ANOVA at particular ages.

Experimental design is shown in Figure 1a. PD14 offspring were housed with their mothers and allowed to feed at will. All offspring of RUPP and Sham pregnancies were weaned on PD21, housed in groups separated by sex with a 12:12 light dark cycle and fed normal rat chow and tap water ad libitum. Fasting animals were placed in a clean cage either singly or with other fasting animals of the same age and sex and only provided tap water. Non‐weaned PD14 rats were returned to dam cages following testing. All blood glucose measurements were determined by glucometer (Bayer, Contour, 9545C, 7097C) via tail snip.

Three glucose homeostasis phenotypes were evaluated: IPGTT (intraperitoneal glucose tolerance test, 2 g glucose/kg); OGTT (oral glucose tolerance test, 5 g glucose/kg); and ITT (insulin tolerance test, 0.75 U insulin/kg). For adult animals (8 Weeks, 6 Months, and 1 Year), a standard overnight fasting time of 16 h was used for IPGTT and OGTT. This fasting time was based on the studies of Intapad et al. (2016) and the literature in rats with the goal of obtaining a stable baseline of blood glucose and insulin levels to manage variability associated with circulating glucose (Bowe et al., 2014). Studies by Nowland et al. (2011) have shown that a 12 or 16 h fast does not increase corticosterone levels in the adult rat, whereas a 24 h fast will. For ITT, all animals were fasted for 6 h to avoid serious hypoglycemia during the test (Benede‐Ubieto et al., 2020). Insulin administration causes a decrease in glucose which can result in seizures or death if the glucose gets too low.

For evaluating glucose tolerance in young PD14 and PD28 animals, we considered that the animal's weight was approximately 35 g and 75 g, respectively, so there was concern that a full 16 h fast would induce a starvation‐like state. Fasting PD28 rats for 12 h resulted in a stable fasting blood glucose like the adult animals (Figure S2). Limited studies are available evaluating fasting times for young rats. Since studies in mice had indicated that a 2–6 h fast stabilized blood glucose, we chose 4 h for our PD14 rats. Fasting blood glucose and weight were measured prior to administration of glucose (Stock 50% dextrose, 0.5 g/mL, NDC 0409‐6648‐16) or insulin (Humalog, Lilly; 100 U/mL). Insulin and glucose dosing was calculated from the fasting weight of the rat. Oral gavage for OGTT used a syringe and flexible 2‐inch plastic gavage needle for PD14 and syringe attached to curved, stainless steel ball‐tipped gavage needle for all other ages. When indicated, blood was collected in heparinized capillary tubes, centrifuged and plasma stored at −80°C for determination of insulin. For analysis of IPGTT and OGTT, area under the curve (AUC) was calculated using the trapezoidal technique with subtraction of the basal glucose as described by Virtue and Vidal‐Puig (2021). The same technique was used to calculate area over the curve (AOC) for ITT with subtraction of the basal glucose.

### 2.3 Glucose stimulated insulin secretion (GSIS) in vitro and in vivo

In vitro evaluation of glucose stimulated insulin secretion was determined as previously described for mice (Lockridge et al., 2020). Islets were isolated from PD13 RUPP and Sham, male and female offspring as previously described (Root et al., 2022). Briefly, pancreata were perfused via the hepatic duct with 0.75 mg/mL ice cold collagenase P (cat no 11213865001; Roche, 1.9 units/mg) in Hanks' Balanced salt solution without calcium chloride, magnesium chloride, or magnesium sulfate (cat no 14174‐095; Gibco). Islets were hand‐picked and cultured overnight with 5 mmol/L glucose. Following a 2‐h incubation in Krebs Buffer with 2 mM glucose (LG), approximately 10 islets were perfused with low glucose (2 mM, 30 min), high glucose (16.7 mM, 30 min), and potassium chloride (30 mM, 15 min). During sequential incubations, supernatant was collected for determination of insulin secretion by ELISA [rat ultrasensitive insulin ELISA per manufacturer's protocol (ALPCO, 80‐INSRTU‐E10)]. Mesh screens containing islets were collected into lysis buffer containing 10% RIPA +1% protease inhibitor cocktail (Abcam; ab201111). DNA of the islets was determined for normalization by Quant‐iT PicoGreen dsDNA assay (Thermo Fisher Scientific, Cat P7589) per kit instructions. Results were expressed as insulin secreted per ng DNA or % insulin content.

For in vivo evaluation of glucose stimulated insulin secretion, PD28 RUPP and Sham female offspring were fasted overnight and then subjected to oral gavage with glucose after fasting as detailed in Methods for OGTT. Approximately 100 μL blood samples were taken, via tail snip, 5–10 min prior to oral gavage, and 10, 20, 30, 60, and 90 min after gavage into heparinized capillary tubes, centrifuged and plasma stored at −80°C for determination of insulin. Insulin was determined by ELISA [rat ultrasensitive insulin ELISA per manufacturer's protocol (ALPCO, 80‐INSRTU‐E01)].

### 2.4 Measurement of pancreatic α and β‐cell ratio, proliferation, and apoptosis

In selected animals, pancreata were isolated, weighed and fixed in 10% neutral buffered formalin overnight and then stored at 4°C in 70% ethanol prior to paraffin embedding. Paraffin‐embedded PD28 rat pancreata were sectioned at 5 μm thickness. Five insulin‐stained sections per animal were taken 200 μm apart through the depth of the pancreas and evaluated. The tissue was deparaffinized using standard CitriSolv (Fisher Scientific 04‐355‐121) and dehydration procedures, followed by pre‐treatment of antigen retrieval, permeabilizing, and blocking as previously described (Akhaphong et al., 2018). For analysis of α and β‐cell areas, the tissue underwent primary staining with rabbit anti‐glucagon (Abcam, ab92517) or guinea pig anti‐insulin (FLEX Guinea Pig Anti‐Insulin, Agilent IR00261‐2) overnight at 4°C, respectively. Several washes of PBS‐0.01% Tween were performed the next day before secondary staining incubation with Cy3 anti‐rabbit antibody (1:500, Jackson ImmunoResearch, Cat 111‐165‐003) or FITC anti‐guinea pig antibody (1:500, Jackson ImmunoResearch, Cat 106‐095‐003) for 90 min at 37°C. After washes of PBS‐0.01% Tween, the slides were dipped in DAPI solution (62248, Thermo Scientific) according to manufacturer's instructions and then cover‐slipped with mounting media (H‐1000, Vector Laboratories). After imaging, α or β‐cell and pancreatic areas were measured to calculate the α or β‐cell ratio, respectively. Images were taken at 10× using a motorized microscope (BZ‐X800E, Keyence). Fiji Software was used to perform image analysis for α or β‐cell ratio. For evaluation of β‐cell proliferation or apoptosis, staining with Ki67 or TUNEL was used as in our previous studies (Root et al., 2022).

### 2.5 Insulin signaling in liver, skeletal muscle, and retroperitoneal fat

After a 6 h fast, PD28 offspring were injected IP with insulin (0.075 U insulin/100 g body weight). Ten minutes after injection, the rat was isoflurane anesthetized and euthanized by pneumothorax. Liver, soleus skeletal muscle, and retroperitoneal fat samples were promptly removed, flash frozen in liquid nitrogen and stored at −80°C until processed. Liver (40–60 mg), retroperitoneal fat (12–58 mg), and soleus muscle (10–45 mg) were homogenized on ice in 200 μL RIPA lysis buffer [1XRIPA buffer (Cell Signaling #9806S), 1% Protease Inhibitor Cocktail EDTA free (Abcam #AB201111), 1% Phosphatase Inhibitor Cocktail I (Abcam AB201112), 1% SDS] for 10–15 s (liver and fat), or 45 s (muscle). Homogenates were agitated 1–2 h (liver and fat) or 2–3 h (muscle) at 4°C then sonicated 3 × 10 s on ice. Sonicated samples were centrifuged for 10 min at 14,000 × *g*. For all 3 tissues, supernatant (avoiding fat and cellular debris layers) was frozen at −80 C until testing. Total protein content of homogenates was determined using BCA (Pierce, Cat. #23337).

For Western blotting, protein samples were denatured and reduced at 95°C for 5 min with 4X NuPAGE LDS Sample Buffer (cat# NP0007) and NuPAGE 10X Reducing Agent (cat# NP0009). For liver and soleus skeletal muscle, 50 μg of total protein was loaded onto Invitrogen Bolt 10% Bis‐Tris Plus WedgeWell™ 1.0 mm 15‐well gels (cat# NW00105BOX), while 25 μg of total protein was loaded onto gels for fat samples. Following SDS‐PAGE, proteins for liver, soleus skeletal muscle, and fat were transferred to PVDF membranes (Amersham™ Hybond™ P Low Fluorescence 0.2 μm #10600102) using BioRad Trans‐blot Turbo Transfer System and blocked for 1 h with 50% Li‐Cor TBS Intercept Blocking Buffer (#927‐6001). Blocked membranes were incubated with primary antibodies, Anti‐Akt (pan) (40D) Mouse mAb (Cell Signaling Technology #2920), Anti‐pAkt (Ser473) (D9E) XP® Rabbit mAb (Cell Signaling Technology #4064), and Anti‐β‐Actin mouse monoclonal (Millipore Sigma #A1978) at dilutions of 1:500, 1:500, and 1:10,000, respectively, in a mixture of Intercept Blocking Buffer, 0.2% Tween20, and 0.02% sodium azide, overnight at 4°C with agitation. Following overnight incubation and washing, secondary antibodies, Li‐Cor IRDye® 800CW Donkey anti‐Rabbit (#925‐32213) and Li‐Cor IRDye® 800CW Donkey anti‐Mouse (#925‐32212), were used at 1:20,000 in a mixture of Li‐Cor Blocking Buffer, 0.2% Tween 20, and 0.015% SDS, and incubated 1 h at room temperature with gentle shaking. Membranes were washed, rinsed, and dried before imaging with Licor Fc Odyssey. Results are expressed as the fold change of the relative density of pAkt/Akt normalized to the respective saline injected control group.

### 2.6 Blood vessel myography

Thoracic aorta was isolated from isoflurane anesthetized male and female PD27‐29 offspring of RUPP or Sham animals. Buffers were prepared according to DMT Multi Wire Myograph System User Manual (Model 620 M; Danish MyoTechnology, Aarhus, Denmark). Segments were placed in ice cold physiological salt solution (PSS) and either cleaned of adipose tissue, or the perivascular adipose tissue (PVAT) was left intact. Segments of 1.5–2 mm length were then mounted on 200 μm hooks and equilibrated for 20–30 min under 10‐12mN resting tension with 2 PSS buffer washes in a Model 620 M DMT myograph. A cumulative phenylephrine concentration response curve was first generated (10^−9^ to 3 × 10^−5^ M). After PSS washing, vessels were precontracted with 10^−7^ M phenylephrine followed by half log increments of acetylcholine (Ach) (5.5 × 10^−10^ to 1.6 × 10^−7^ M) to assess endothelial‐dependent relaxation. After PSS washing, vessels were again contracted with 10^−7^ M phenylephrine and relaxation to cumulative insulin (Humulin R 100 U/mL) was assessed (1.2, 3.6, 12 μM/0.2, 0.6, 2 Units/mL). After washing with PSS and return to baseline tension, 10^−7^ M phenylephrine precontraction was followed by relaxation to increasing concentrations of sodium nitroprusside (SNP; 1.2 × 10^−10^ to 1.2 × 10^−6^ M) as a measure of endothelial‐independent vasodilation. Finally, exposure to high potassium (60 mM potassium in KPSS buffer) confirmed maximal vessel reactivity.

### 2.7 Statistical analysis

**FIGURE 2:**
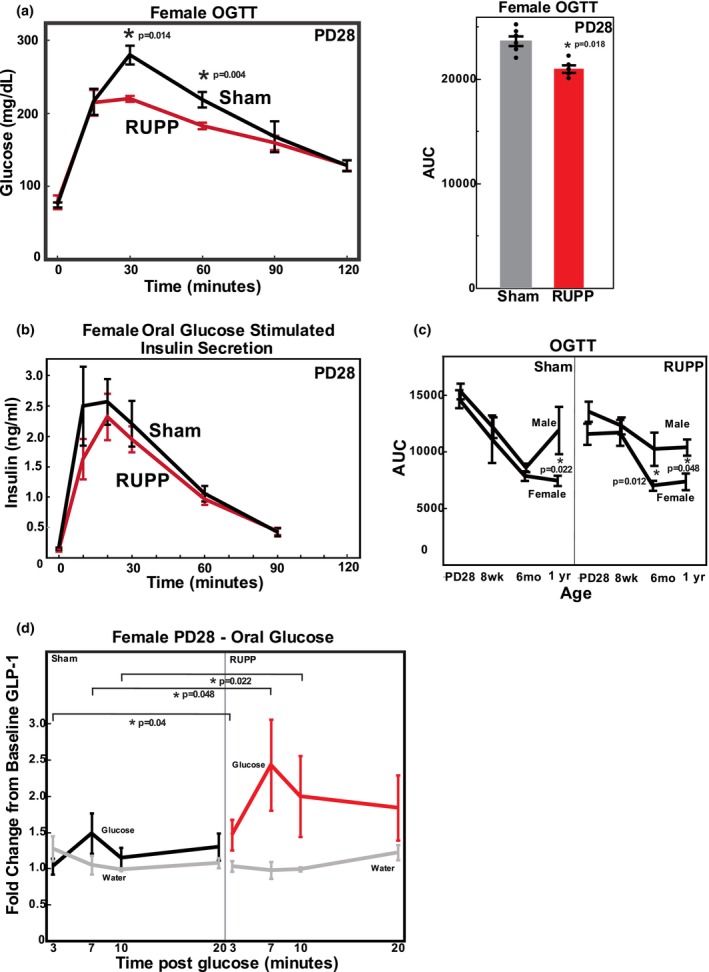
Oral glucose tolerance testing (OGTT) of Sham and RUPP offspring. (a) Female PD28 offspring challenged with oral glucose. Area under the curve (AUC) from time 0 glucose was calculated using 15, 30, 60, 90, and 120 min. Values represent mean +/− SE from 5 to 6 offspring from different litters. *Significantly different for AUC or at individual timepoint comparing Sham and RUPP. (b) Plasma insulin measurements in PD28 female offspring after oral glucose administration. Values represent the mean+/− SE from 7 to 8 offspring from different litters. (c) AUC after OGTT at different ages of male and female offspring from Sham and RUPP pregnancies. AUC was calculated for all ages and sexes of offspring using 30, 60, 90, and 120 min time points as seen in Figure S4. Values represent mean +/− SE from 5 to 13 offspring of each sex from different litters. In select groups, two pups of the same sex from a litter were included (Sham Female PD28, *n* = 6; RUPP Male PD28, *n* = 6; Sham Male 8 Weeks, *n* = 9). ANOVA detected a significant sex (*p* = 0.0011) and age (*p* = 0.0001) effect overall. *p values less than 0.05 are indicated for individual contrast following ANOVA. (d) Fold change in plasma active GLP‐1 determined by ELISA at 3, 7, 10, and 20 min following oral challenge of PD28 female offspring with either glucose or water. Animals were treated with 10 mg/kg sitagliptin and baseline GLP‐1 determined 15 min prior to glucose or water gavage. Values represent the mean +/− SE from 5 to 7 offspring from different litters. **Significantly different comparing GLP‐1 from glucose challenged Sham to glucose challenged RUPP offspring, *p* values less than 0.05 are indicated.

**FIGURE 3:**
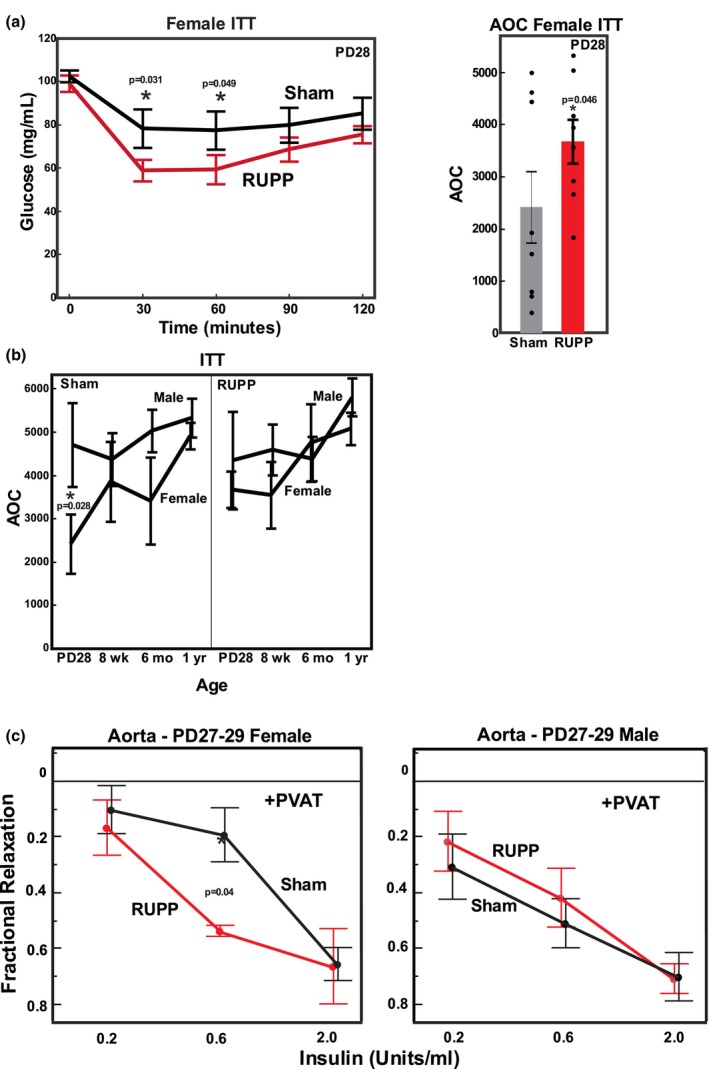
Insulin tolerance of offspring from Sham and RUPP pregnancies. (a) Female PD28 offspring were fasted and challenged by ip injection of insulin. Glucose was determined by glucometer from tail bleeds. Area over the curve from time 0 glucose was calculated using 30, 60, 90, and 120 min. Values represent mean +/− SE from 8 offspring from different litters. *p values less than 0.05 indicated for surgery effect with AOC or for individual contrast at each time point following ANOVA. (b) Male and female offspring from Sham and RUPP pregnancies were challenged by IP injection of insulin after fasting. Glucose was determined by glucometer and Area over the curve (AOC) from time 0 glucose was calculated for PD28, 8 week, 6 month, and 1 year offspring using 30, 60, 90, and 120 min time points for all ages and both sexes as seen in Figure S6. Values represent mean +/− SE from 5 to 13 offspring of each sex from different litters. In select groups, two pups of the same sex from a litter were included (RUPP Male 8 Weeks, *n* = 15; Sham Male 8 Weeks, *n* = 15; RUPP Male 6 Month, *n* = 10; Sham Male 6 Month, *n* = 8; RUPP Male 1 year, *n* = 11). ANOVA detected a significant sex (*p* = 0.015) and age (*p* = 0.014) effect overall. *p values less than 0.05 indicated for individual contrast at PD28 comparing male and female following ANOVA. (c) Fractional relaxation to insulin in thoracic aorta from male and female PD27‐29 offspring of Sham and RUPP pregnancies. Repeated measures ANOVA was conducted with insulin dose, surgery, and interactions followed by post hoc comparisons at individual insulin doses comparing RUPP and Sham. Values represent mean +/− SE from 4 to 7 offspring of each sex from different litters. *p values less than 0.05 are indicated for individual contrast at each dose.

**FIGURE 4:**
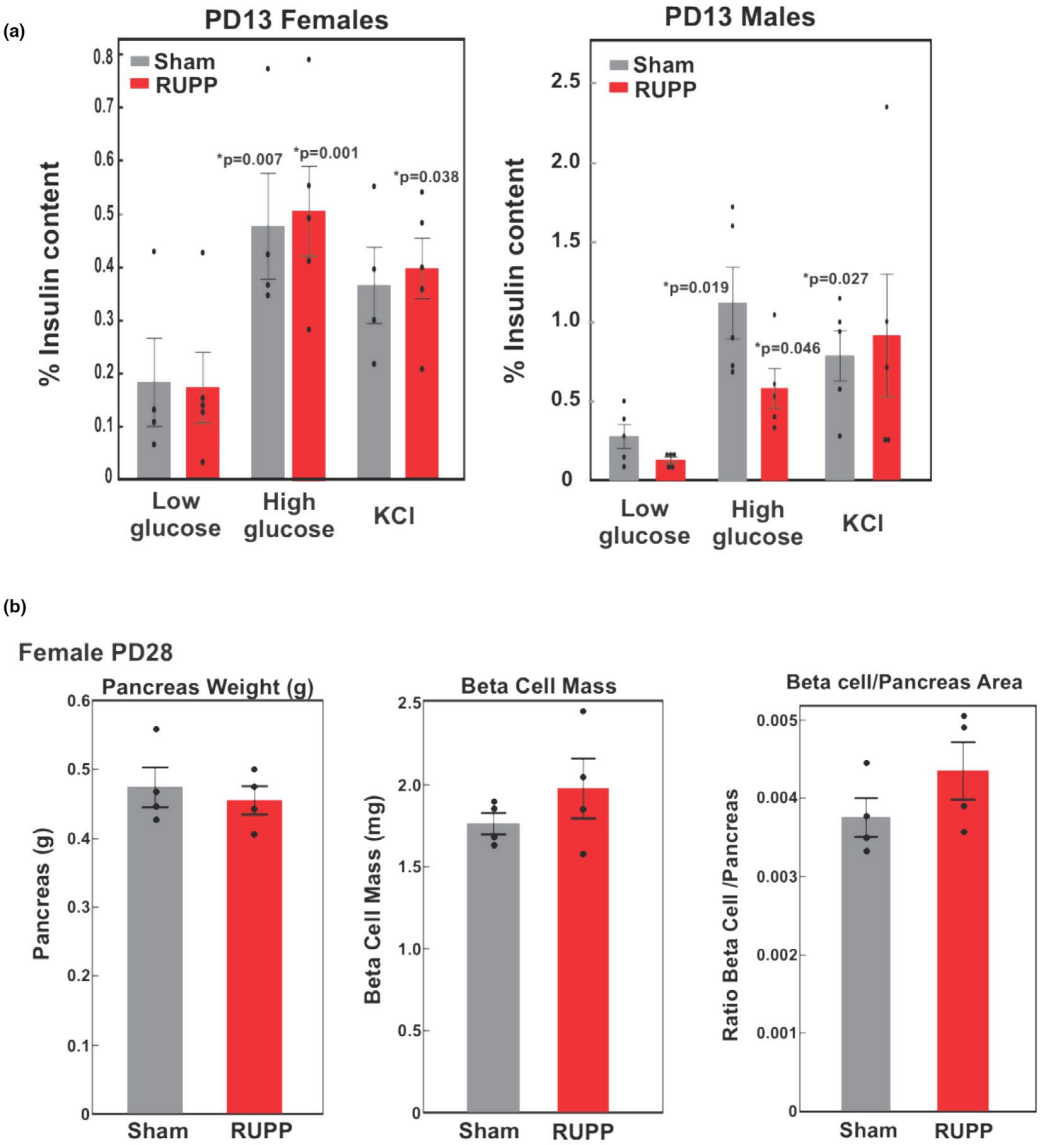
Glucose stimulated insulin secretion from PD13 isolated islets and PD28 Beta cell mass. (a) Islets were isolated from PD13 female and male, Sham and RUPP offspring and exposed sequentially to 30 min of low glucose (2 mM), high glucose (16.7 mM), and KCl (30 mM). Supernatant was collected for insulin determination and expressed as % insulin content for normalization of data. Values represent the mean +/− SE from islets isolated from 4 to 5 offspring of each sex from different litters. *p < 0.05 compared to respective low glucose treatment. (b) β‐cell/pancreas area was determined by image analysis of paraffin embedded sections of pancreas stained for insulin in PD28 female offspring of dams undergoing either Sham or RUPP surgery. Values represent the mean +/− SE from 4 female offspring from different litters.

For each end point we used one male and one female pup from each litter with the following exceptions. In three groups for OGTT, two offspring of the same sex from one of the litters were used (Sham Female PD28, *n* = 6; RUPP Male PD28, *n* = 6; Sham Male 8 Weeks, *n* = 9) and this is indicated in the figure legend (Figure 2, Figure S4). In five groups for ITT, two offspring of the same sex from one of the litters were used (RUPP Male 8 Weeks, *n* = 15; Sham Male 8 Weeks, *n* = 15; RUPP Male 6 Month, *n* = 10; Sham Male 6 Month, *n* = 8; RUPP Male 1 year, *n* = 11) and this is indicated in the figure legend (Figure 3, Figure S6). Values in figures are presented as Mean +/− SEM. Table S1 includes mean, SD, and SEM for all data in the figures. Two‐tailed Student's *t*‐test was used for data comparing α and β‐cell/pancreas area (Figure 4b). Two‐way ANOVA using individual offspring values was used for other analyses with sex, surgery, and interactions considered, both for analysis of glucose phenotypes over time as well as the AOC/AUC analysis. Comparing male and female RUPP and Sham Offspring, post hoc comparisons considered were as follows: Sham female versus RUPP female, Sham male versus RUPP male, Sham female versus Sham male. For analysis of aortic relaxation to insulin, repeated measures ANOVA was used considering insulin dose, surgery, and interactions, followed by post hoc comparisons at each insulin dose in RUPP versus Sham. All statistical analysis used JMP and SAS software (SAS Institute, Cary, NC) with *p* < 0.05 considered significant.

### 2.8 GLP‐1 assay

Biologically active GLP‐1 [GLP‐1 (7–36 amide) and GLP‐1 (7‐37)] was measured with EMD Millipore GLP‐1 (Active) ELISA kit (cat#EGLP‐35K). PD28 rats were gavaged with sitagliptin (Sigma‐Aldrich, PHR1857‐1G) dissolved in water at 10 mg/kg body weight based on the studies of Thomas et al. (2008). After 30 min, a baseline sample was obtained, followed 15 min later by either glucose (5 g Dextrose/kg body weight) or water vehicle gavage. Tail blood samples were collected at 3, 7, 10, and 20 min post‐glucose or water gavage. Tail blood samples of 160 μL were collected into chilled EDTA coated capillary tubes (80 μL MiniCollect®, Greiner Bio‐one ref#450434) and immediately mixed with DPP‐IV Inhibitor (EMD Millipore Catalog # DPP4) at 10 μL/mL blood, and 3 mM EDTA and kept on ice. Samples were centrifuged for 10 min at 1000 × *g*. Plasma was removed and again centrifuged 10 min at 1000 × *g* to remove any residual cellular components. Plasma samples were stored at −80°C until testing.

## 3 RESULTS

### 3.1 Body weight of RUPP and sham offspring

Our previous studies had demonstrated a reduced fetal weight at e19 in RUPP versus Sham pregnancies (Akhaphong et al., 2018) with a minor but significant decrease in body weight still demonstrable at PD13 in male and female RUPP compared to Sham offspring (Root et al., 2022). Similarly, at PD28, body weight of female, but not male offspring was slightly less in RUPP offspring compared to Sham (Figure S1). With age, weight gain was equivalent throughout the first year comparing RUPP and Sham of the same sex from 8 weeks through 1 year. Male offspring weighed significantly more than female offspring at all ages evaluated (Figure S1).

### 3.2 Changes in glucose tolerance in aging RUPP and sham offspring

We had previously reported that 8–9 week old female offspring of RUPP pregnancies did not exhibit altered glucose tolerance compared to Sham offspring following intraperitoneal glucose administration (Root et al., 2022) despite having reduced β‐cell area at PD13. Since studies by Alexander et al. (Intapad et al., 2016, 2019) had reported changes in oral glucose tolerance in RUPP versus Sham offspring at 6 months and 1 year of age, we expanded our studies to evaluate glucose tolerance, both IP and with oral glucose administration through the first year of age. Fasting blood glucose in all age groups was no different in RUPP versus Sham offspring (Figure S2). Male offspring had higher fasting blood glucose than female at 1 year of age along with a tendency for a difference at 6 months of age. With IP administration of glucose (IPGTT), the response in RUPP and Sham offspring did not differ with either sex at PD14, PD28, 8 weeks, 6 months, and 1 year (Figure S3, Figure 1b). However, sex and age differences were detected, and this is best illustrated by examining AUC for male and female, Sham and RUPP offspring in the different age groups (Figure 1c). In Figure 1c, AUC for IPGTT was calculated using the 30 through 120 min time points for all ages. AUC increased with age, with a greater increase in males versus females. The sex effect was significant at 6 months and 1 year with changes in response to IP glucose being more prolonged in male offspring compared to females as reflected in the increased AUC from the baseline (Figure 1c, Figure S3).

In contrast to IP administration, oral glucose administration (OGTT) resulted in a significant difference in glucose tolerance in RUPP offspring compared to Sham at PD28 but not at other ages (Figure S4; Figure 2a). To illustrate the sex differences over the age groups, the AUC for male and female, Sham and RUPP offspring was calculated using 15 through 120 min time points (Figure 2c). A significant sex and age effect was noted, with the female offspring more effectively clearing glucose, particularly at 1 year, whether RUPP or Sham. The OGTT AUC significantly decreased with age.

### 3.3 Glucose stimulated insulin secretion in RUPP and sham offspring

To probe the mechanism of the change in glucose tolerance following oral administration of glucose (Figure 2a), we hypothesized that RUPP offspring produced more insulin due to an enhanced incretin effect through GLP‐1 compared to Sham offspring. Thus, we measured both circulating insulin and GLP‐1 following oral glucose administration. As seen in Figure 2b, following oral glucose administration to PD28 female offspring, circulating insulin was no different in RUPP versus Sham offspring. For GLP‐1, initial experiments showed relatively minor changes in active GLP‐1 in PD28 female offspring with oral glucose administration, despite collection of tail blood samples into EDTA and dipeptidase inhibitor. To ensure that degradation of active GLP‐1 was being prevented, animals were administered the dipeptidase inhibitor sitagliptin 45 min prior to oral glucose administration. With sitagliptin treatment in vivo, active GLP‐1 significantly increased following oral glucose administration, compared to water administration as a control (Figure 2d). The fold change in GLP‐1 was greater and more sustained in RUPP PD28 female offspring than Sham (Figure 2d).

Given that circulating insulin did not increase to a greater extent in RUPP versus Sham female offspring following oral glucose (Figure 2b), but GLP‐1 did (Figure 2d), we evaluated glucose stimulated insulin secretion in isolated islets from PD13 offspring where a reduction in β‐cell area had clearly been demonstrated (Root et al., 2022). Islets isolated from PD13 offspring showed significant increase in insulin when stimulated with high glucose or potassium. However, RUPP and Sham offspring did not differ in the amount of insulin secretion whether stimulated with low or high glucose or maximally with potassium chloride (Figure 4a). Thus, glucose stimulated insulin secretion in vitro (Figure 4a) at PD13 or in vivo (Figure 2b) at PD28 did not differ in RUPP or Sham offspring.

### 3.4 α‐cell and β‐cell area, proliferation, and apoptosis

We also considered that changes in α‐ or β‐cell area might contribute to changes in the glucose phenotype. Our previous studies demonstrated a significant decrease in β‐cell area and an increase in β‐cell apoptosis in the e19 fetus and the PD13 pups from RUPP versus Sham pregnancies (Akhaphong et al., 2018; Root et al., 2022). In addition, at PD13 we noted a decrease in β‐cell proliferation, islet size, and β‐cell size. In view of the change in oral glucose tolerance observed at PD28 in female offspring, we evaluated β‐cell area or mass, proliferation and apoptosis in female RUPP and Sham offspring. No detectable difference in β‐cell area was apparent (Figure 4b). In addition, the β‐cell proliferation and apoptotic rate were back to Sham values at PD28 (Figure S5). Evaluation of α cell area in the PD28 islets also showed no difference (Figure S5). Thus, these data suggest that compensatory mechanisms had restored the β‐cell area/mass in female RUPP offspring by PD28 along with improving glucose tolerance (Figure 2a).

### 3.5 Changes in insulin tolerance in aging RUPP and sham offspring

Given that the improvement in oral glucose tolerance is not associated with increased insulin production in RUPP offspring compared to Sham, we evaluated insulin sensitivity of peripheral tissues in all age groups (Figure S6; Figure 3a) with differences detected comparing RUPP versus Sham only in PD28 female offspring. In Figure 3a, AOC for ITT was calculated using the 30 through 120 min time points for all ages as described (Virtue & Vidal‐Puig, 2021). A significant surgery effect (RUPP vs. Sham) was seen in the PD28 RUPP female offspring, but not in other age groups. Sensitivity to insulin was greater in RUPP compared to Sham offspring as evidenced by changes in the time course and an increase in AOC (Figure 3a). ANOVA analysis of the ITT data in all surgery, sex, and age groups (Figure 3b) revealed significant sex and age effects (*p* < 0.05, Figure 3b). Individual comparisons of sex at each age (Figure 3b, Figure S6) demonstrated that PD28 Sham females were less sensitive to insulin than the Sham males.

Increased insulin sensitivity in female RUPP offspring at PD28 could be due to numerous factors including enhanced insulin induced vasodilation leading to improved glucose delivery to sites of glucose uptake. We tested this in the PD28 offspring by assessing the ability of insulin to relax a thoracic aorta segment that was pre‐contracted with a submaximal dose of phenylephrine. Previous studies of others had demonstrated that insulin‐induced vasodilation was increased in the presence of perivascular adipose tissue (PVAT), so vasodilation of aorta was tested with and without PVAT. Vasodilation in response to insulin was more evident in aorta segments with PVAT still present, whether male or female, (Figure 3c, Figure S7) consistent with published studies of others in the mouse (Meijer et al., 2013). In the presence of PVAT, aorta from female RUPP offspring had enhanced fractional relaxation to insulin, that is, vasodilation compared to female Sham offspring. These data suggest that enhanced delivery of glucose to peripheral tissues may in part explain the increased insulin sensitivity in female RUPP offspring seen in Figure 3a.

**FIGURE 5:**
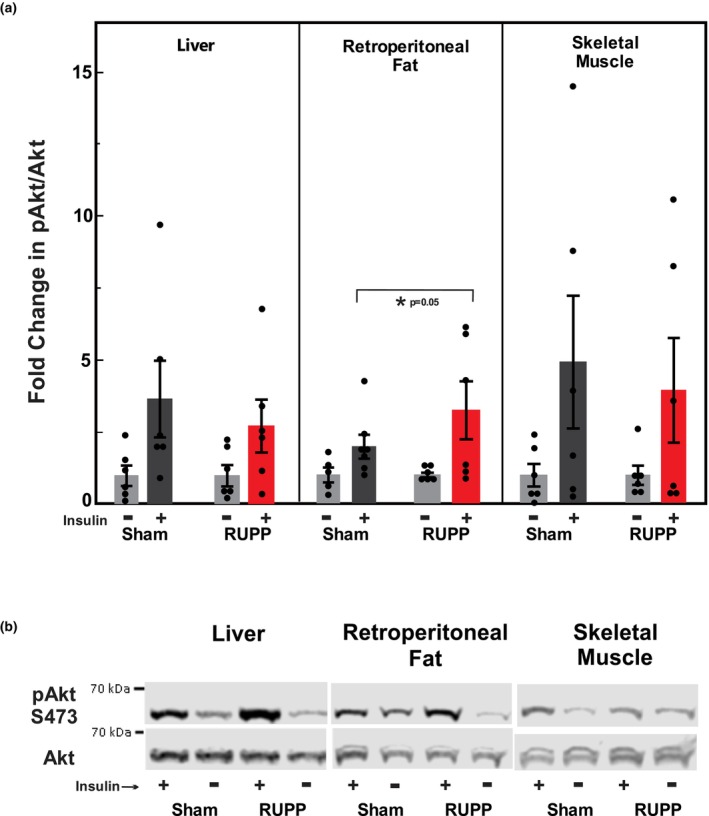
Insulin signaling. (a) Phosphorylation of Akt in liver, retroperitoneal fat, and skeletal muscle obtained from PD28 Sham or RUPP female offspring 10 min after injection with either saline (−) or insulin (+). Animals were fasted 6 h prior to injection. Values represent the mean +/− SE of the fold change in pAkt/Akt band density compared to the respective saline injected control in 5–7 offspring from different litters. Insulin significantly increased pAkt in all tissues. *Significant increase compared to Sham challenged with insulin with a *p* value of 0.05. (b) Representative Western blots for pAkt and Akt in liver, retroperitoneal fat, and skeletal muscle (soleus). Each lane represents pAkt and Akt from the same animal sample.

Increased insulin sensitivity in female RUPP offspring at PD28 could also involve increased insulin signaling in the liver, skeletal muscle, and/or fat as evidenced by increased phosphorylation of Akt at serine 473. Insulin stimulation significantly increased pAkt in liver, soleus muscle, and retroperitoneal fat of PD28 female offspring compared to a control injection of insulin vehicle (saline) (Figure 5a,b, Figure S8). Akt phosphorylation after insulin challenge was significantly greater in the retroperitoneal fat of PD28 RUPP offspring compared to Sham, but not in the liver or skeletal muscle. These data suggest that increased insulin sensitivity in the peripheral fat may in part explain the increased response to insulin seen in RUPP offspring in the insulin tolerance test. Total body fat was not assessed in these animals. However, we dissected and weighed the total retroperitoneal fat from PD27‐29 Sham and RUPP, male and female offspring. Female PD27‐29 RUPP offspring had more retroperitoneal fat than Sham animals, and male had more than female (*n* = 4–11: Sham Female, 0.13 ± 0.02 g; RUPP Female, 0.22 ± 0.02; Sham Male, 0.18 ± 0.01; RUPP Male, 0.29 ± 0.03).

## 4 DISCUSSION

Developmental programming of T2D following gestational hypertension and IUGR is a major health concern. Clearly, insults in utero can severely impact health of the offspring. However, our studies demonstrate compensatory mechanisms that come to play in protecting the growth restricted offspring of a hypertensive pregnancy from long term metabolic effects and the development of T2D. Despite a hypertensive pregnancy with growth restriction and reduced β‐cell area at early offspring ages, the female offspring of a RUPP pregnancy recovered normal β‐cell area by PD28 and were able to effectively handle glucose and retain or enhance insulin sensitivity with aging. The improved oral glucose tolerance in female RUPP offspring was associated with a greater increase in the incretin GLP‐1 compared to Sham offspring. The improved insulin sensitivity in RUPP offspring coincided with enhanced vasodilation to insulin and increased insulin‐induced phosphorylation of Akt in fat, suggesting improved delivery of glucose to peripheral tissues. These findings point to the importance of the incretins in regulating glucose handling and the potential of insulin vasodilation to affect overall insulin sensitivity.

Since Intapad et al. (2016) had seen a difference in OGTT in offspring of RUPP rats, we evaluated the effect of an oral glucose challenge and anticipated seeing a difference in glucose tolerance in RUPP versus Sham by this route. Instead of observing an increased AUC in RUPP offspring compared to Sham, we noted a decrease. Small et al. (2022) did a detailed analysis of differences in response to glucose by the intraperitoneal versus oral route in mice and found that intraperitoneal administration results in elevated blood glucose but a largely absent elevation in insulin and incretins when compared to oral administration. Thus, we investigated an enhanced role for insulin or GLP secretion in the RUPP PD28 offspring compared to Sham. We found a greater increase in GLP‐1 in female RUPP offspring at PD28 when oral glucose challenge resulted in a suppression of the glucose response in the circulation. However, glucose stimulated insulin secretion in vivo did not differ in PD28 female RUPP offspring compared to Sham, when increased GLP‐1 was evident. This suggests that the increased GLP‐1 is improving glucose tolerance in an insulin independent manner. GLP‐1 has also been reported to cause relaxation of the rat aorta (Green et al., 2008) through a GLP receptor and cAMP, independent of the endothelium. GLP‐1 also increases frequency and force of contraction of the heart. Centrally it can decrease food intake and appetite. Thus, GLP‐1 has known effects beyond its ability to increase insulin and can act centrally as well as in numerous peripheral tissues to affect glucose regulation and metabolism (Folli et al., 2023; Muller et al., 2019; Nauck & Meier, 2018; Zhao et al., 2021).

Our previous studies demonstrated reduced β‐cell area at PD13 characterized by reduced proliferation and increased apoptosis (Akhaphong et al., 2018). This effect was attributable to smaller or fewer islets per area of pancreas. Our previous studies at PD13 had not evaluated changes in insulin secretion in isolated islets. We considered that a change in islet insulin secretion at PD13 might influence the glucose handling evident at PD28. However, glucose stimulated insulin secretion in islets from PD13 RUPP offspring was not different than Sham. In adult mouse islets, KCl is known to induce a stronger and more immediate insulin secretion response than glucose alone. Our data in PD13 islets showed a similar effect on insulin when stimulated with high glucose and KCl. This has also been observed by others when using neonatal rat islets (Yang et al., 2021). The design of our experiments is also important to consider, where islets were treated with high glucose for 30 min and only 15 min with KCl. KCl, as a depolarizing agent triggers rapid insulin secretion, while glucose stimulates a more metabolically driven and sustained insulin release, thus the longer glucose exposure may have contributed to the relative amount of insulin produced.

At PD28, we found the α‐ and β‐cell area of RUPP were unchanged compared to Sham, and the β‐cell area, proliferation, and apoptosis are back to normal. Other models of intrauterine growth restriction such as the low‐protein diet during pregnancy cause a β‐cell mass deficit at birth that is recovered by young adulthood (Alejandro et al., 2014). Studies of others have shown that in the face of decreased β‐cell area, α‐cells secrete GLP‐1, and that GLP‐1 is a β‐cell mitogen and antiapoptotic (Holter et al., 2022; Zheng et al., 2024). From our measurement of active GLP‐1 following oral glucose challenge, we know that RUPP PD28 female offspring can produce more GLP‐1 than Sham offspring, and this may be operating to restore β‐cell area.

Restoration of β‐cell area associated with restoration of normal β‐cell proliferation and increased β‐cell size in RUPP offspring was also accomplished between e19 and PD13 by macrophage depletion with clodronate liposomes (Root et al., 2022). These data suggested that changes in macrophage populations in islets play a critical role in restoring normal β‐cell proliferation and might account for the return to normal β‐cell area. Thus, numerous factors are in play in vivo influencing the β‐cell area and the likelihood of developing glucose intolerance and insulin resistance.

The recovery of β‐cell/pancreas area at PD28 may be due to reduced apoptosis, increased proliferation, or hypertrophy of β‐cells in the period between PD13 and PD28. In a study by Scaglia et al. (1997), β‐cell mass significantly increased in Sprague Dawley rats from PD20 to PD31, with a rapid increase in pancreas weight during a period between PD17 and PD24. Islet neogenesis was also observed at PD13 and later, with the number of β‐cells nearly doubling between PD20 and PD24. Similarly, in studies by Bonner‐Weir et al. (2016, 2012), 30%–50% of new β‐cells observed during the first month of life in the rat were attributed to neogenesis. Another study by Dor et al. (2004) suggests that β‐cells can self‐duplicate and thus the necessity for stem cell differentiation in β‐cell replenishment is unclear. In addition, β‐cell regeneration in rodents and humans may be different (Zhong & Jiang, 2019) with clear changes in the replicative ability of β‐cells over the lifespan. Our studies have not addressed how the β‐cell area is restored at PD28 but will be the subject of future investigations.

A clear difference in insulin‐induced vasodilation in PD28 offspring from RUPP pregnancies compared to Sham was demonstrated in our study. Perivascular adipose tissue (PVAT) has been shown to control vascular tone in aorta and other vessels in response to a variety of contractile agonists. In addition, PVAT unmasks insulin‐induced vasodilation in mouse gracilis muscle (Meijer et al., 2013). Marchesi et al. (2009) demonstrated that PVAT decreased the contractile response to norepinephrine, endothelin and angiotensin II in mesenteric arteries of mice. Insulin induced vasodilation has been studied using cumulative concentration response curves in rat aorta (Crissey et al., 2015). In our study, insulin clearly caused a greater vasodilation (fractional relaxation to acetylcholine) of the thoracic aorta from RUPP versus Sham offspring in the presence of PVAT suggesting that insulin generated in the RUPP offspring following glucose challenge would dilate blood vessels and potentially cause more efficient delivery of glucose to peripheral tissues for uptake into fat, skeletal muscle, and liver. These studies are limited using a conducting artery rather than an artery supplying major fat or skeletal muscle such as used by Meijer et al. (2013). In addition, high concentrations of insulin were needed for vasodilation, even in the presence of PVAT.

Increased Akt phosphorylation, suggesting increased insulin signaling, was noted in the PD28 RUPP offspring compared to Sham indicating that part of the increased insulin sensitivity may be related to glucose removal by the peripheral fat. In a study by Ozanne et al. (1996), male offspring from dams fed a low protein diet during pregnancy and lactation had increased insulin sensitivity and glucose uptake in skeletal muscle, along with more insulin receptors compared to offspring from dams fed a high protein diet during pregnancy and lactation. In contrast, Intapad reported decreased insulin sensitivity in the periphery in RUPP offspring at older ages (Intapad et al., 2016).

Sex‐dependent differences in IPGTT, OGTT, and ITT are evident from our studies evaluating the AUC or AOC for glucose tolerance or insulin sensitivity. With IPGTT and OGTT, the AUC was less for females than males, consistent with human studies and increased glucose tolerance in female versus male mice seen in our recent study (Jo et al., 2023). For insulin sensitivity, a significant sex effect (Figure 3b) indicated that females were generally less sensitive to insulin when all age groups were considered, with the most marked difference at PD28 when comparing Sham males to females. As the Sham animals aged, differences in insulin sensitivity between the sexes was less apparent. In our recent study comparing insulin sensitivity in normal adult mice, greater insulin sensitivity was apparent in female versus male mice (Jo et al., 2023) similar to observations in humans (Gannon et al., 2018). This may reflect a species difference since a study by Vital et al. in rats also noted reduced insulin sensitivity in female Wistar rats compared to male (Vital et al., 2006).

The basis for the sex difference in altered β‐cell area and change in glucose phenotypes is unknown. The sex difference in β‐cell area was evident at e19 and PD13 in the RUPP offspring, and in the significant sex effects observed in glucose phenotypes. Intapad et al. explored sex difference in their studies of RUPP offspring where they had observed glucose intolerance in 6 and 12 month female offspring but not male. In their study, ovariectomy enhanced the glucose intolerance at 6 and 12 months of age in the female offspring (Intapad et al., 2016), and testosterone was protective in male RUPP offspring (Intapad et al., 2017) suggesting a hormonal effect was operative. In rats, estrogen is high in both male and females through weaning, whereas after weaning, estrogen, and testosterone concentrations begin to increase into adulthood (Bell, 2018). At PD28, male testosterone levels begin to increase but female testosterone changes are significantly lower throughout development in females. Further investigation in the developmental origins of disease in both sexes in utero is warranted.

Age‐dependent differences were also evident from our studies where glucose tolerance decreased with age when glucose was administered intraperitoneally. However, when glucose was administered orally, glucose tolerance increased with age suggesting that incretins were a bigger factor in controlling glucose as the animal aged. Insulin sensitivity also changed with age, with older animals more responsive to the ability of insulin to decrease blood glucose.

Studies by others have shown that alterations in placental blood supply can lead to changes in β‐cell area and to glucose intolerance in offspring. In a study by Simmons et al. (2001), complete ligation of the uterine arteries on GD19 of a 21.5 day gestation in pregnant rats, resulted in more severe IUGR than our study, with approximately a 1 g difference in ligated versus control birth weights and a reduced β‐cell mass reported at 15 and 26 weeks for offspring. They clearly demonstrated glucose intolerance at 7 weeks of age whereas our studies did not. The Simmons study differed from our study in the severity of the ligation and greater fetal growth restriction noted. Unlike our study, they also used normal females to nurse offspring from both control and uterine ligation treatment groups, thus controlling for the influence of the maternal environment after birth. Offspring sex was not considered in their analyses, and they only observed changes in β‐cell mass at 15 and 26 weeks of age (not at 1 and 7 weeks), whereas we detected decreased β‐cell area much earlier at e19 and PD13. Simmons also reported insulin resistance in IUGR offspring as early as 1 week and continuing through 15 weeks. In our current study, RUPP offspring were not insulin resistant, and in fact PD28 female offspring were more sensitive to insulin than Sham offspring. In the study by Simmons, blood pressure elevation after complete ligation of the uterine arteries was not determined, where we have previously reported significant hypertension in our reduced uteroplacental perfusion pressure model.

At PD28, the female RUPP offspring weighed slightly but significantly less than the Sham offspring, yet the weight of their retroperitoneal fat was greater. This imbalance between body weight and visceral fat may contribute to altered regulation of glucose levels in offspring of a hypertensive pregnancy. Certainly the increased retroperitoneal fat could be due to increased insulin signaling in the peripheral fat leading to increased glucose uptake in PD28 offspring and increased fat storage (Ludwig & Ebbeling, 2018). Simmons also reported increased fat pad weights in growth restricted offspring following complete uterine ligation (Simmons et al., 2001).

Intapad et al. (2016) reported that female offspring of a RUPP pregnancy were glucose intolerant at 6 month and 1 year of age following an oral glucose challenge. In contrast to the study of Intapad, we did not demonstrate glucose intolerance and in fact PD28 IUGR offspring more effectively controlled blood glucose compared to Sham offspring. Intapad saw no changes in insulin sensitivity in IUGR offspring whereas we saw greater insulin sensitivity in PD28 female offspring. Intapad also reported a reduction in insulin signaling [IRβ (an insulin receptor) and GLUT4 (an insulin dependent glucose transporter)] in white adipose tissue of IUGR animals at 12 months. However, we reported increased phosphorylation of Akt in the retroperitoneal fat of PD28 female IUGR offspring compared to Sham, suggesting increased insulin signaling is contributing to the phenotype observed at this developmental stage in the rat. In the study by Intapad, β‐cell numbers were not measured. Intapad's studies primarily differed from ours in the severity of the IUGR where they reported approximately a gram difference in birthweight compared to our 0.1–0.2 g difference (Akhaphong et al., 2018; Root et al., 2022). The drastic difference in birthweight in our study compared to theirs could be due to subtle differences in the RUPP technique between labs. They clearly report a much larger difference in birthweight that could potentially have more lasting effects in the developing offspring. Another difference between our studies and those of Intapad et al. is the source of the Sprague Dawley rat that could account for some of the differences reported. Numerous studies have noted conflicting results comparing Sprague Dawley rats obtained from Charles Rivers versus Harlan/Envigo (Reckelhoff & Alexander, 2018) pointing to the importance of considering rat supplier, breeding techniques and husbandry conditions when results differ across studies.

In summary, our studies revealed an improvement in the ability of offspring to handle glucose following a hypertensive pregnancy with IUGR. Our studies also revealed compensatory mechanisms operating to reverse changes in β‐cell area following an in‐utero insult and point to the plasticity of the β‐cell during development.

## AUTHOR CONTRIBUTIONS

KMR, MAC, EUA, and JFR designed the experiments. JFR, MAC, KMR, and AEM performed the animal studies. BA determined the α and β‐cell mass. MB determined glucose stimulated insulin secretion from isolated islets. JFR and RRR performed the primary analysis of the data. JFR, MAC, KMR, AEM, and EUA drafted the manuscript which was reviewed, edited, and approved by all the authors. EUA and JFR are the guarantors of this work and, as such, had full access to all the data in the study and take responsibility for the integrity of the data and the accuracy of the data analysis.

## FUNDING INFORMATION

NIH R21 HD100840 (EUA and JFR). Whiteside Institute for Clinical Research, a collaboration of St. Luke's Hospital, Clinics and Foundation and the University of Minnesota Medical School, Duluth Campus (JFR).

## ETHICS STATEMENT

All procedures were approved by the Institutional Animal Care and Use Committee of the University of Minnesota and conformed to National Institutes of Health guidelines.

## CONFLICT OF INTEREST STATEMENT

No potential or perceived conflicts of interest relevant to this article to disclose.

## Supporting information


Appendix S1.

## Data Availability

Data generated in the current study are available from the corresponding authors on reasonable request.
