# Supplementary material for: Improved glucose handling in female rat offspring of a hypertensive pregnancy with intrauterine growth restriction
Source: Physiol Rep. 2025 Feb 4;13(3):e70222. doi: 10.14814/phy2.70222 (PMC11792987; doi:10.14814/phy2.70222)
Supplement: Supplementary file 1 — Appendix S1. [file PHY2-13-e70222-s001.docx]

**SUPPLEMENTAL MATERIAL**

**Improved Glucose Handling in Female Rat Offspring of a Hypertensive Pregnancy with Intrauterine Growth Restriction**

Melissa A. Cedars^1#^, Kate M. Root^1#^, Brian Akhaphong^2^, Megan Beetch ^2^, Abigail E. Miles^1^, Ronald R. Regal^3^, Emilyn U. Alejandro^2^, Jean F. Regal^1^

^1^Department of Biomedical Sciences, University of Minnesota Medical School, Duluth, Minnesota 55812, USA

^2^Department of Integrative Biology and Physiology, University of Minnesota Medical School, Minneapolis, MN 55455, USA

^3^ Department of Mathematics and Statistics, University of Minnesota, Duluth, Minnesota 55812 USA

^#^Equal contributors as first authors

**Co-corresponding authors:**

Jean F. Regal, Ph.D.

Department of Biomedical Sciences

University of Minnesota Medical School, Duluth Campus

1035 University Dr.

Duluth, MN 55812

Tel: 218 726 8950

Fax: 218 726 7906

Email: jregal@d.umn.edu

Emilyn U. Alejandro, Ph.D.

Department of Integrative Biology and Physiology

University of Minnesota Medical School

Minneapolis, MN 55455, USA

Tel: 612-626-7768

Email: [ealejand@umn.edu](mailto:ealejand@umn.edu)

**Supplemental Fig S1**


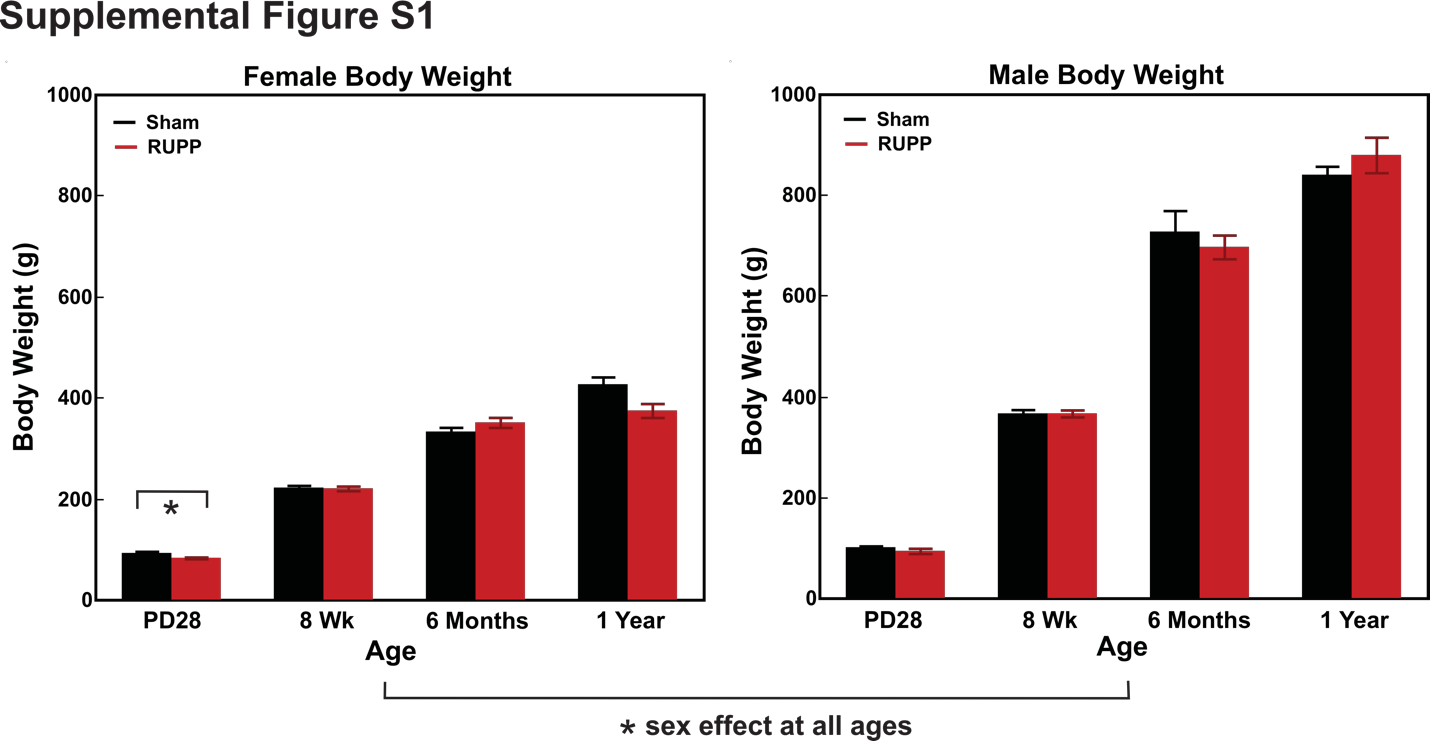


**Supplemental Fig S1. Body weight of male and female Sham and RUPP offspring across the first year.** Body weight was determined after 6 hour fast and prior to ITT. Values represent the mean +/- SE. * indicates p<0.05 for sex effect following ANOVA. For PD28 female animals, * indicates p<0.05 comparing RUPP to Sham female offspring. N=6-11 offspring of each sex from different litters.

**Supplemental Fig S2**


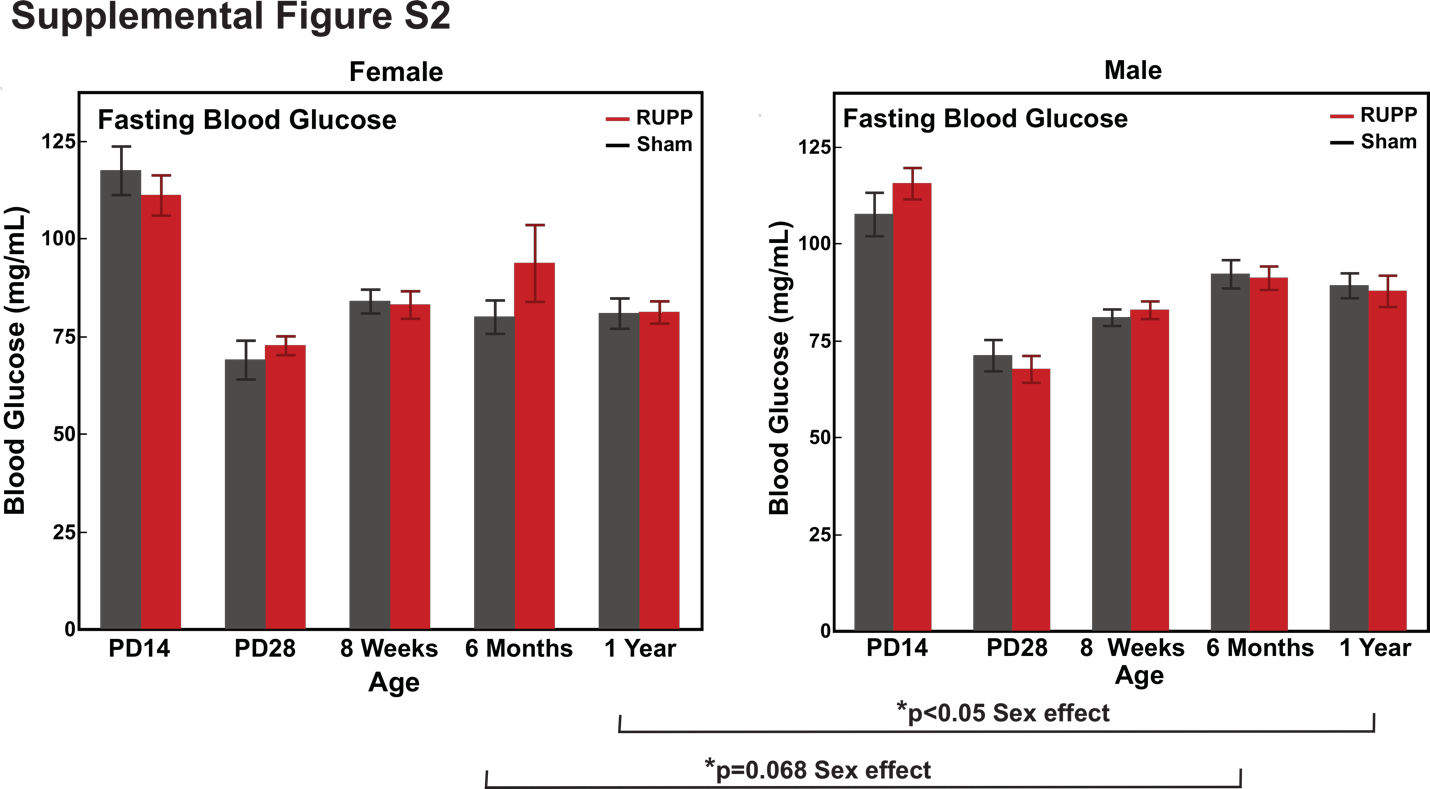


**Supplemental Fig S2. Fasting blood glucose in male and female offspring from Sham and RUPP.** Blood glucose was determined prior to IPGTT testing with fasting as described in Methods. Values represent the mean +/- SE. * indicates p<0.05 for sex effect following ANOVA. N=6-11 offspring of each sex from different litters.

**Supplemental Fig S3**


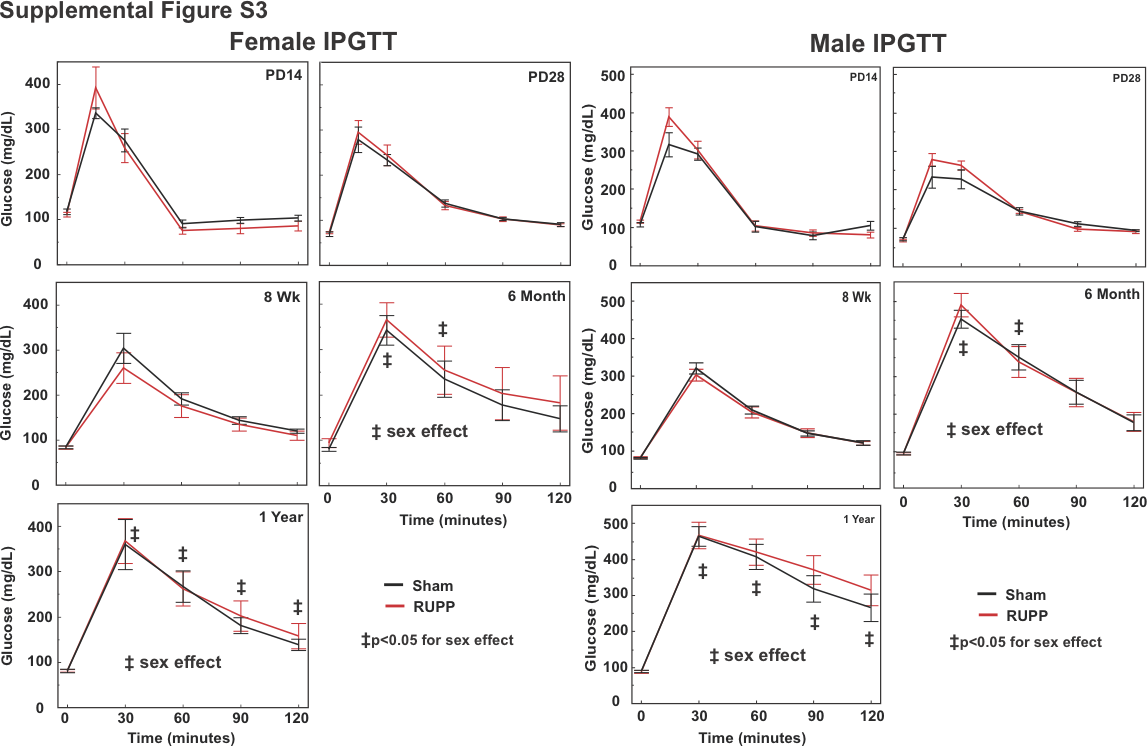


**Supplemental Fig S3. IPGTT in female and male Sham and RUPP offspring from PD14 to 1 year of age.** Offspring from Sham and RUPP pregnancies were challenged IP with glucose after fasting as described in Methods. Values represent mean +/- SE from 6-11 offspring of each sex from different litters. ANOVA evaluated sex, surgery and interactions at each time point with post hoc comparisons. No significant surgery effect was detected. ‡indicates p<0.05 for a sex effect at the time points indicated.

**Supplemental Fig S4**


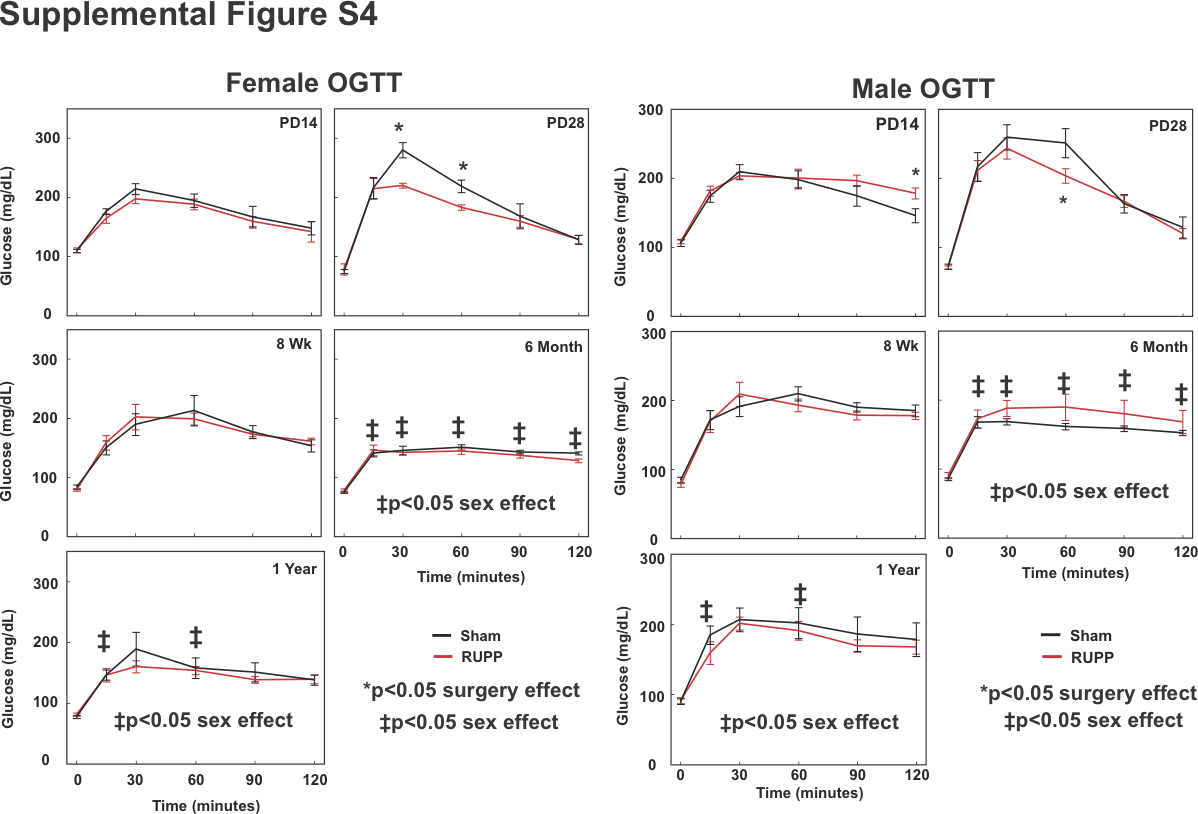


**Supplemental Fig S4.** **OGTT in female and male Sham and RUPP offspring from PD14 to 1 year of age**. Offspring from Sham and RUPP pregnancies were challenged by oral gavage with glucose in fasted animals as described in Methods. Values represent mean +/- SE from 5-13 offspring of each sex from different litters. In select groups, 2 pups of the same sex from a litter were included (Sham Female PD28, n=6; RUPP Male PD28, n=6; Sham Male 8 Wk, n=9). ANOVA evaluated sex, surgery and interactions at each time point with post hoc comparisons. *indicates p<0.05 for a surgery effect at the indicated time point. ‡indicates p<0.05 for a sex effect at the time points indicated.

**Supplemental Fig S5**


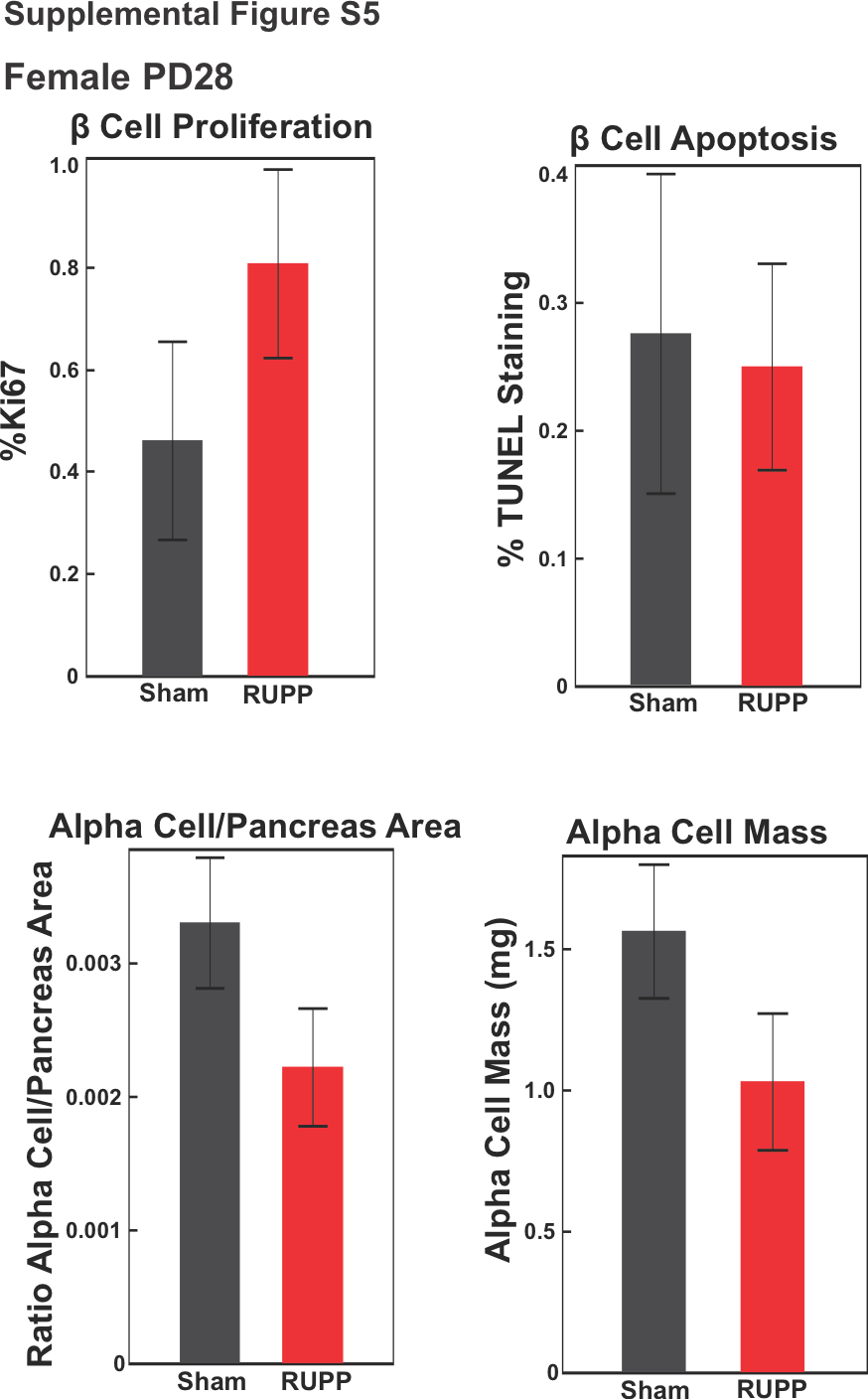


Supplemental Fig S5. **Changes in β-cell proliferation and apoptosis as well as alpha cell area in pancreas of female PD28 offspring of Sham and RUPP pregnancies.** Values represent mean +/- SE from 4 female offspring from different Sham or RUPP litters. No difference between RUPP and Sham was detected in any of the outcomes using Student’s t test.

**Supplemental Fig S6**


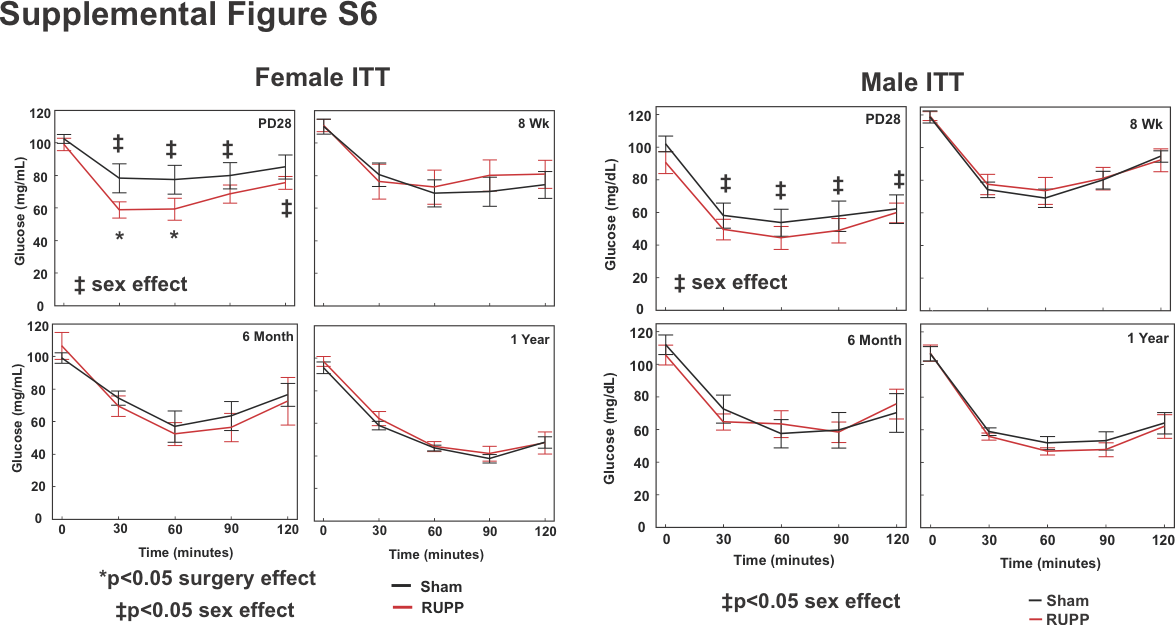


**Supplemental Fig S6. ITT in female and male Sham and RUPP offspring from PD28 to 1 year of age.** Offspring from Sham and RUPP pregnancies were challenged by IP administration of insulin as described in Methods. Values represent mean +/- SE from 7-15 offspring of each sex from different litters. In select groups, 2 pups of the same sex from a litter were included (RUPP Male 8 Wk, n=15; Sham Male 8 Wk, n=15; RUPP Male 6 Month, n=10; Sham Male 6 Month, n=8; RUPP Male 1 Year, n=11). ANOVA evaluated sex, surgery and interactions at each time point with post hoc comparisons. *indicates p<0.05 for a surgery effect at the indicated time point. ‡indicates p<0.05 for a sex effect at the time points indicated.

**Supplemental Fig S7**


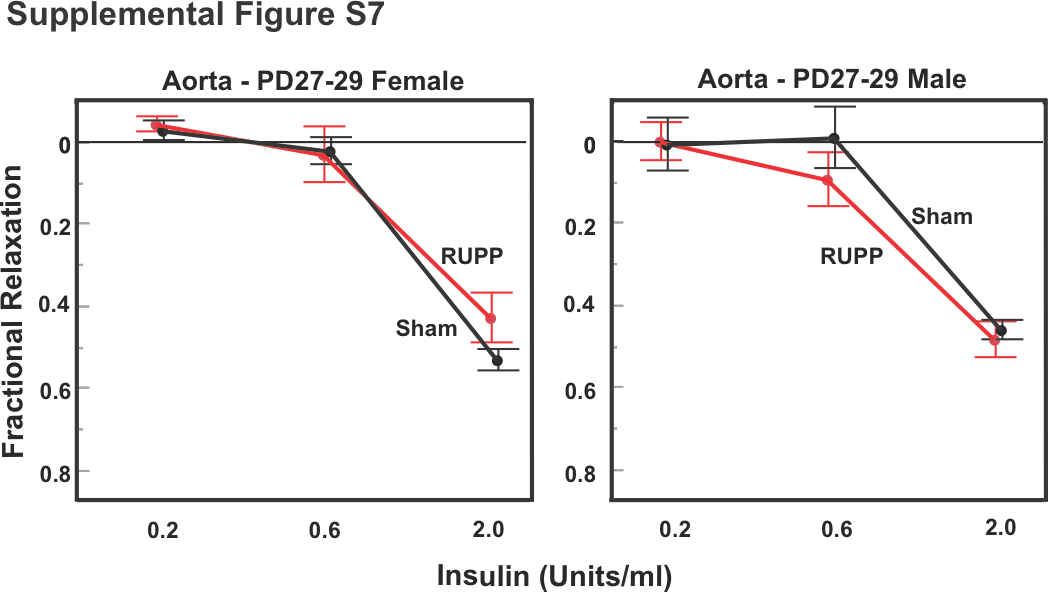


**Supplemental Fig S7. Fractional relaxation to insulin in thoracic aorta from female and male PD27-29 offspring of Sham and RUPP pregnancies.** Perivascular adipose tissue was removed, aortas were pre-contracted with phenylephrine and the relaxation to increasing doses of insulin determined. Values represent mean +/- SE from 4-7 offspring of each sex from different litters.

Supplemental Fig S8

**Supplemental Fig S8.** Complete representative Western blots for pAkt and Akt in retroperitoneal fat from RUPP and Sham offspring (Animals A-F) challenged with insulin or saline with portions (Animals C-F) shown in Fig 5B. Each lane represents pAkt and Akt from the same animal sample run in duplicate on one gel, with one half of the gel probed with pAkt antibody and the other half probed with Akt antibody.

| **Supplemental Table 1** | |  |  |  |
| --- | --- | --- | --- | --- |
|  |  |  |  |  |
| Figure 1B-IPGTT Female PD28 | | |  |  |
|  |  |  |  |  |
| Sham |  |  |  |  |
|  | Mean | SD | SE | N |
| 0 min | 69 | 12.18 | 4.97 | 6 |
| 15 min | 278.33 | 69.2 | 28.25 | 6 |
| 30 min | 233.33 | 30.33 | 12.38 | 6 |
| 60 min | 137.17 | 19.55 | 7.98 | 6 |
| 90 min | 102.67 | 7.89 | 3.22 | 6 |
| 120 min | 90.5 | 10.6 | 4.33 | 6 |
|  |  |  |  |  |
| RUPP |  |  |  |  |
|  | Mean | SD | SE | N |
| 0 min | 72.67 | 5.92 | 2.42 | 6 |
| 15 min | 294.67 | 64.35 | 26.27 | 6 |
| 30 min | 243.83 | 56.27 | 22.97 | 6 |
| 60 min | 132.5 | 22.12 | 9.03 | 6 |
| 90 min | 102.33 | 12.63 | 5.16 | 6 |
| 120 min | 89.17 | 7.83 | 3.2 | 6 |

| Figure 1C-IPGTT Area Under the Curve | | |  |  |
| --- | --- | --- | --- | --- |
|  |  |  |  |  |
| Sham Female |  |  |  |  |
|  | Mean | SD | SE | N |
| PD28 | 7388.57 | 2883.32 | 1089.79 | 7 |
| 8 wk | 12128.18 | 5295.61 | 1596.69 | 11 |
| 6 mo | 16456.88 | 10051.82 | 3553.85 | 8 |
| 1 yr | 17829 | 10548.62 | 3335.77 | 10 |
|  |  |  |  |  |
| Sham Male |  |  |  |  |
|  | Mean | SD | SE | N |
| PD28 | 8377.5 | 4257.63 | 1346.38 | 10 |
| 8 wk | 13670.29 | 3361.68 | 815.33 | 17 |
| 6 mo | 24570 | 7634.79 | 2544.93 | 9 |
| 1 yr | 30286.25 | 10806.51 | 3119.57 | 12 |
|  |  |  |  |  |
| RUPP Female |  |  |  |  |
|  | Mean | SD | SE | N |
| PD28 | 8067.5 | 2305.35 | 941.15 | 6 |
| 8 wk | 10015.5 | 6649.52 | 2102.76 | 10 |
| 6 mo | 17580 | 11913.63 | 4212.1 | 8 |
| 1 yr | 18793.5 | 11654.01 | 3685.32 | 10 |
|  |  |  |  |  |
| RUPP Male |  |  |  |  |
|  | Mean | SD | SE | N |
| PD28 | 9343.33 | 1056.23 | 352.08 | 9 |
| 8 wk | 12752.81 | 4806.8 | 1201.7 | 16 |
| 6 mo | 25438.33 | 10401.89 | 3467.3 | 9 |
| 1 yr | 33213.41 | 11368.78 | 3427.82 | 11 |

| Figure 2A-OGTT Female PD28 | | |  |  |  | Figure 2A-OGTT Female PD28 Area Under the Curve | | | | |
| --- | --- | --- | --- | --- | --- | --- | --- | --- | --- | --- |
|  |  |  |  |  |  |  |  |  |  |  |
| Sham |  |  |  |  |  | Sham |  |  |  |  |
|  | Mean | SD | SE | N |  |  | Mean | SD | SE | N |
| 0 min | 74.83 | 8.42 | 3.44 | 6 |  |  | 14658.75 | 1942.46 | 793.01 | 6 |
| 15 min | 216 | 43.81 | 17.88 | 6 |  |  |  |  |  |  |
| 30 min | 280 | 31.43 | 12.83 | 6 |  | RUPP |  |  |  |  |
| 60 min | 218.83 | 25.97 | 10.6 | 6 |  |  | Mean | SD | SE | N |
| 90 min | 168.17 | 51.79 | 21.14 | 6 |  |  | 11559 | 2094.78 | 936.81 | 5 |
| 120 min | 128.5 | 18.62 | 7.6 | 6 |  |  |  |  |  |  |
|  |  |  |  |  |  |  |  |  |  |  |
| RUPP |  |  |  |  |  |  |  |  |  |  |
|  | Mean | SD | SE | N |  |  |  |  |  |  |
| 0 min | 78.4 | 21.01 | 9.39 | 5 |  |  |  |  |  |  |
| 15 min | 214.6 | 39.16 | 17.51 | 5 |  |  |  |  |  |  |
| 30 min | 220 | 9.11 | 4.07 | 5 |  |  |  |  |  |  |
| 60 min | 183 | 10.12 | 4.53 | 5 |  |  |  |  |  |  |
| 90 min | 159.6 | 21.94 | 9.81 | 5 |  |  |  |  |  |  |
| 120 min | 128.8 | 15.55 | 6.95 | 5 |  |  |  |  |  |  |

| Figure 2B-OGTT GSIS Female PD28 | | |  |  |
| --- | --- | --- | --- | --- |
|  |  |  |  |  |
| Sham |  |  |  |  |
|  | Mean | SD | SE | N |
| 0 min | 0.15 | 0.05 | 0.02 | 8 |
| 10 min | 2.5 | 1.84 | 0.65 | 8 |
| 20 min | 2.57 | 1.06 | 0.38 | 8 |
| 30 min | 2.21 | 1.06 | 0.38 | 8 |
| 60 min | 1.06 | 0.36 | 0.13 | 8 |
| 90 min | 0.42 | 0.19 | 0.07 | 8 |
|  |  |  |  |  |
| RUPP |  |  |  |  |
|  | Mean | SD | SE | N |
| 0 min | 0.14 | 0.04 | 0.01 | 7 |
| 10 min | 1.7 | 0.99 | 0.37 | 7 |
| 20 min | 2.38 | 1.15 | 0.43 | 7 |
| 30 min | 1.83 | 0.53 | 0.2 | 7 |
| 60 min | 0.96 | 0.24 | 0.09 | 7 |
| 90 min | 0.47 | 0.15 | 0.06 | 7 |

| Figure 2C-OGTT Area Under the Curve | | |  |  |
| --- | --- | --- | --- | --- |
|  |  |  |  |  |
| Sham Female |  |  |  |  |
|  | Mean | SD | SE | N |
| PD28 | 14658.75 | 1942.46 | 793.01 | 6 |
| 8 wk | 11120 | 5142.15 | 2099.28 | 6 |
| 6 mo | 7862.73 | 1411.87 | 425.69 | 11 |
| 1 yr | 7440 | 1194.16 | 451.35 | 7 |
|  |  |  |  |  |
| Sham Male |  |  |  |  |
|  | Mean | SD | SE | N |
| PD28 | 15340.5 | 1547.81 | 692.2 | 5 |
| 8 wk | 12140 | 2744 | 914.67 | 9 |
| 6 mo | 8590.38 | 1360.4 | 377.31 | 13 |
| 1 yr | 11890.57 | 6938.67 | 2092.09 | 11 |
|  |  |  |  |  |
| RUPP Female |  |  |  |  |
|  | Mean | SD | SE | N |
| PD28 | 11559 | 2094.78 | 936.81 | 5 |
| 8 wk | 11587.5 | 2303.86 | 940.55 | 6 |
| 6 mo | 7008 | 1400.1 | 442.75 | 10 |
| 1 yr | 7487.81 | 1844.86 | 652.26 | 8 |
|  |  |  |  |  |
| RUPP Male |  |  |  |  |
|  | Mean | SD | SE | N |
| PD28 | 13550 | 2174.51 | 887.74 | 6 |
| 8 wk | 12295.71 | 1982.08 | 749.16 | 7 |
| 6 mo | 10236.25 | 5098.78 | 1471.89 | 12 |
| 1 yr | 10385.36 | 1892.254 | 715.2 | 7 |

| Figure 2D-OGTT GLP Female PD28 | | |  |  |
| --- | --- | --- | --- | --- |
|  |  |  |  |  |
| Sham Glucose |  |  |  |  |
|  | Mean | SD | SE | N |
| 3 min | 0.96 | 0.25 | 0.1 | 6 |
| 7 min | 1.59 | 0.76 | 0.31 | 6 |
| 10 min | 1.17 | 0.35 | 0.14 | 6 |
| 20 min | 1.38 | 0.49 | 0.2 | 6 |
|  |  |  |  |  |
| Sham Water |  |  |  |  |
|  | Mean | SD | SE | N |
| 3 min | 1.28 | 0.38 | 0.17 | 5 |
| 7 min | 0.97 | 0.28 | 0.13 | 5 |
| 10 min | 0.91 | 0.16 | 0.07 | 5 |
| 20 min | 0.95 | 0.28 | 0.12 | 5 |
|  |  |  |  |  |
| RUPP Glucose |  |  |  |  |
|  | Mean | SD | SE | N |
| 3 min | 1.51 | 0.88 | 0.33 | 7 |
| 7 min | 2.65 | 2.24 | 0.85 | 7 |
| 10 min | 2.14 | 1.66 | 0.63 | 7 |
| 20 min | 2 | 1.52 | 0.57 | 7 |
|  |  |  |  |  |
| RUPP Water |  |  |  |  |
|  | Mean | SD | SE | N |
| 3 min | 1.06 | 0.16 | 0.07 | 5 |
| 7 min | 1 | 0.25 | 0.11 | 5 |
| 10 min | 0.99 | 0.06 | 0.03 | 5 |
| 20 min | 1.29 | 0.38 | 0.17 | 5 |

| Figure 3A-In vitro GSIS PD13 Islets | | |  |  |  |  |  |  |  |  |
| --- | --- | --- | --- | --- | --- | --- | --- | --- | --- | --- |
|  |  |  |  |  |  |  |  |  |  |  |
| Female Sham |  |  |  |  |  | Male Sham |  |  |  |  |
|  | Mean | SD | SE | N |  |  | Mean | SD | SE | N |
| Low Glucose | 0.1846 | 0.1658 | 0.0829 | 4 |  | Low Glucose | 0.2831 | 0.1688 | 0.0755 | 5 |
| High Glucose | 0.4767 | 0.1998 | 0.0999 | 4 |  | High Glucose | 1.1264 | 0.5017 | 0.2243 | 5 |
| KCl | 0.3657 | 0.1434 | 0.0717 | 4 |  | KCl | 0.7854 | 0.3519 | 0.1574 | 5 |
|  |  |  |  |  |  |  |  |  |  |  |
| Female RUPP |  |  |  |  |  | Male RUPP |  |  |  |  |
|  | Mean | SD | SE | N |  |  | Mean | SD | SE | N |
| Low Glucose | 0.1719 | 0.1486 | 0.0664 | 5 |  | Low Glucose | 0.1348 | 0.0441 | 0.0197 | 5 |
| High Glucose | 0.5096 | 0.1886 | 0.0844 | 5 |  | High Glucose | 0.5845 | 0.2776 | 0.1241 | 5 |
| KCl | 0.3972 | 0.1277 | 0.0571 | 5 |  | KCl | 0.9092 | 0.8607 | 0.3849 | 5 |

| Figure 3B-Pancreas and Beta-cell Female PD28 | | | |  |
| --- | --- | --- | --- | --- |
|  |  |  |  |  |
| Pancreas Weight |  |  |  |  |
|  | Mean | SD | SE | N |
| Sham | 0.4741 | 0.0582 | 0.0291 | 4 |
| RUPP | 0.455 | 0.0408 | 0.0204 | 4 |
|  |  |  |  |  |
| Beta Cell Mass |  |  |  |  |
|  | Mean | SD | SE | N |
| Sham | 1.7622 | 0.1299 | 0.0649 | 4 |
| RUPP | 1.9767 | 0.3665 | 0.1832 | 4 |
|  |  |  |  |  |
| Beta cell/Pancreas Area | |  |  |  |
|  | Mean | SD | SE | N |
| Sham | 0.00375 | 0.0005 | 0.00025 | 4 |
| RUPP | 0.00435 | 0.00073 | 0.00037 | 4 |

| Figure 4A-ITT Female PD28 | | |  |  |  | Figure 4A-ITT Female PD28 Area Over the Curve | | | | |
| --- | --- | --- | --- | --- | --- | --- | --- | --- | --- | --- |
|  |  |  |  |  |  |  |  |  |  |  |
| Sham |  |  |  |  |  | Sham |  |  |  |  |
|  | Mean | SD | SE | N |  |  | Mean | SD | SE | N |
| 0 min | 102.5 | 7.73 | 2.73 | 8 |  |  | 2415 | 1933.48 | 683.59 | 8 |
| 30 min | 78.31 | 25.21 | 8.91 | 8 |  |  |  |  |  |  |
| 60 min | 77.44 | 25.02 | 8.85 | 8 |  | RUPP |  |  |  |  |
| 90 min | 79.88 | 22.83 | 8.07 | 8 |  |  | Mean | SD | SE | N |
| 120 min | 85.25 | 20.93 | 7.4 | 8 |  |  | 3671.25 | 1185.33 | 419.08 | 8 |
|  |  |  |  |  |  |  |  |  |  |  |
| RUPP |  |  |  |  |  |  |  |  |  |  |
|  | Mean | SD | SE | N |  |  |  |  |  |  |
| 0 min | 99.13 | 10.75 | 3.8 | 8 |  |  |  |  |  |  |
| 30 min | 58.88 | 14.12 | 4.99 | 8 |  |  |  |  |  |  |
| 60 min | 59.31 | 19.09 | 6.75 | 8 |  |  |  |  |  |  |
| 90 min | 68.63 | 15.74 | 5.56 | 8 |  |  |  |  |  |  |
| 120 min | 75.5 | 11.16 | 3.95 | 8 |  |  |  |  |  |  |

| Figure 4B-ITT Area Over the Curve | | |  |  |
| --- | --- | --- | --- | --- |
|  |  |  |  |  |
| Sham Female |  |  |  |  |
|  | Mean | SD | SE | N |
| PD28 | 2415 | 1933.48 | 683.59 | 8 |
| 8 wk | 3853.64 | 3059.85 | 922.58 | 11 |
| 6 mo | 3410.63 | 2841.34 | 1004.56 | 8 |
| 1 yr | 4909.5 | 969.18 | 306.48 | 10 |
|  |  |  |  |  |
| Sham Male |  |  |  |  |
|  | Mean | SD | SE | N |
| PD28 | 4703.06 | 2738.74 | 968.29 | 8 |
| 8 wk | 4368 | 2363.63 | 610.29 | 15 |
| 6 mo | 5026.88 | 1386.47 | 490.19 | 8 |
| 1 yr | 5325 | 1553.79 | 448.54 | 12 |
|  |  |  |  |  |
| RUPP Female |  |  |  |  |
|  | Mean | SD | SE | N |
| PD28 | 3671.25 | 1185.33 | 419.08 | 8 |
| 8 wk | 3543.75 | 2174.15 | 768.68 | 8 |
| 6 mo | 4748.57 | 2367.13 | 894.69 | 7 |
| 1 yr | 5074.5 | 1184.7 | 374.63 | 10 |
|  |  |  |  |  |
| RUPP Male |  |  |  |  |
|  | Mean | SD | SE | N |
| PD28 | 4342.5 | 2979.84 | 1126.27 | 7 |
| 8 wk | 4588.93 | 2195.69 | 586.82 | 14 |
| 6 mo | 4378.5 | 1616.1 | 511.06 | 10 |
| 1 yr | 5796.82 | 1450.78 | 437.43 | 11 |

| Figure 4C-Fractional Relaxation PD27-29 Aorta | | | |  |  |  |  |  |  |  |
| --- | --- | --- | --- | --- | --- | --- | --- | --- | --- | --- |
|  |  |  |  |  |  |  |  |  |  |  |
| Female Sham |  |  |  |  |  | Male Sham |  |  |  |  |
|  | Mean | SD | SE | N |  |  | Mean | SD | SE | N |
| 0.2 U/mL | 0.1468 | 0.2201 | 0.0898 | 6 |  | 0.2 U/mL | 0.2464 | 0.246 | 0.142 | 3 |
| 0.6 U/mL | 0.284 | 0.287 | 0.1172 | 6 |  | 0.6 U/mL | 0.4427 | 0.1391 | 0.0803 | 3 |
| 2 U/mL | 0.6837 | 0.156 | 0.0637 | 6 |  | 2 U/mL | 0.643 | 0.1557 | 0.0899 | 3 |
|  |  |  |  |  |  |  |  |  |  |  |
| Female RUPP |  |  |  |  |  | Male RUPP |  |  |  |  |
|  | Mean | SD | SE | N |  |  | Mean | SD | SE | N |
| 0.2 U/mL | 0.1441 | 0.2498 | 0.1249 | 4 |  | 0.2 U/mL | 0.1778 | 0.2338 | 0.0955 | 6 |
| 0.6 U/mL | 0.553 | 0.0316 | 0.0158 | 4 |  | 0.6 U/mL | 0.3601 | 0.2547 | 0.104 | 6 |
| 2 U/mL | 0.6022 | 0.3096 | 0.1548 | 4 |  | 2 U/mL | 0.6901 | 0.1163 | 0.0475 | 6 |

| Figure 5A-pAKT/Akt Female PD28 Liver, Fat, and Muscle | | | | |
| --- | --- | --- | --- | --- |
|  |  |  |  |  |
| Liver |  |  |  |  |
|  | Mean | SD | SE | N |
| Sham - | 1 | 0.87 | 0.35 | 6 |
| Sham + | 3.66 | 3.26 | 1.33 | 6 |
| RUPP - | 1 | 0.91 | 0.37 | 6 |
| RUPP + | 2.72 | 2.25 | 0.92 | 6 |
|  |  |  |  |  |
| Retroperitoneal Fat | |  |  |  |
|  | Mean | SD | SE | N |
| Sham - | 1 | 0.58 | 0.26 | 5 |
| Sham + | 1.98 | 1.09 | 0.41 | 7 |
| RUPP - | 1 | 0.19 | 0.08 | 6 |
| RUPP + | 3.24 | 2.45 | 1 | 6 |
|  |  |  |  |  |
| Skeletal Muscle |  |  |  |  |
|  | Mean | SD | SE | N |
| Sham - | 1 | 0.98 | 0.4 | 6 |
| Sham + | 5.02 | 5.78 | 2.36 | 6 |
| RUPP - | 1 | 0.84 | 0.34 | 6 |
| RUPP + | 4.02 | 4.56 | 1.86 | 6 |
